# Optimized placement and sizing of solar photovoltaic distributed generation using jellyfish search algorithm for enhanced power system performance

**DOI:** 10.1038/s41598-025-08227-4

**Published:** 2025-07-01

**Authors:** P Rajakumar, P. M. Balasubramaniam, E. Parimalasundar, K. Suresh, P. Aravind

**Affiliations:** 1 Vel Tech Rangarajan Dr.Sagunthala R&D Institute of Science and Technology, Chennai, Tamil Nadu India; 2 Department of ECE, Hindusthan Institute of Technology, Coimbatore, Tamil Nadu India; 3 Mohan Babu University, Tirupati, India; 4 Christ (Deemed to be University), Bangalore, India; 5 Mattu University, Mattu, Ethiopia

**Keywords:** Metaheuristic optimization, Jellyfish search algorithm, Distributed generation, Voltage deviation, Voltage stability, Photovoltaic, Power loss, Engineering, Materials science

## Abstract

The strategic integration of distributed generation (DG) units into distribution power networks (DPNs) is pivotal for augmenting system efficiency and stability. This study introduces an advanced metaheuristic optimization framework leveraging the Jellyfish Search Algorithm (JSA) for the optimal placement and sizing of solar photovoltaic (PV) DG units. The formulated multi-objective function incorporates real power loss (RPL) minimization, voltage deviation index (VDI) reduction, and voltage stability index (VSI) enhancement, employing a weighted sum approach (WSA) to ensure computational rigor. The efficacy of the proposed methodology is rigorously validated on the IEEE 33-bus radial DPN under single and multiple PV system deployment scenarios. For single PV system optimized inclusion, RPL of the DPN is cut down from 210.98 kW to 102.89 kW, total VDI is reduced from 1.8047 p.u to 0.5331 p.u, and minimum VSI is increased from 0.6671 to 0.7559. For two PV DG units inclusion, RPL is reduced to 82.99 kW, total VDI is reduced to 0.6518 p.u with a least VSI improved to 0.8848. However, better result is obtained with three units of DG placement with RPL reduced to 69.59 kW, total VDI decreased to 0.3293 p.u with a least VSI of the test system increased to 0.8916. Comparative analyses against state-of-the-art metaheuristic algorithms underscore the superior convergence efficiency and optimality of JSA in addressing nonlinearity and high-dimensionality constraints. Empirical results substantiate substantial RPL reduction, bus voltage enhancement, and system stability reinforcement, establishing JSA as an avant-garde paradigm in DG optimization.

## Introduction

Power system utilities supply electricity through transmission and distribution power networks. The consumers are powered through distribution power networks (DPN). DPN connects the consumers through low-potential and high-current conductors. The power losses (PL) and voltage drops along the DPN are higher than those of the transmission networks (TN), since the line resistance of the DPN is higher than that of the TN. High PL, low voltage, frequent interruptions, harmonic distortions (current and voltage), and unstable voltage limit the performance of DPN. Hence, DPN performance should be optimized for reliable and stable operation. Various approaches, including capacitor assimilation, distributed generation (DG) placement, network reconfiguration, and FACTS device placement have been adopted to increase the performance of DPN^1,2^. Capacitors and few FACTS such as SVC (Static VAR Compensator), STATCOM (Static Synchronous Compensator) and UPFC (Unified Power Flow Controller) inject sufficient quantities of reactive power to minimize PL and regulate bus voltages of DPN. The network reconfiguration method enhances the performance of DPN by changing the tie-line and sectionalized switches. DG is a decentralized generation system that uses modern PV technology, wind turbines or microturbines to inject real (P), reactive (Q), or both into the DPN. DG optimization is considered more effective than capacitor, SVC, STATCOM and UPFC since it can support the DPN with both real and reactive power injection. Integration of DG changes the existing radial DPN into an active system. DG can be defined as a typical small-scale power generator^3^. Moreover, renewable energy resource (RER) based DGs are highly recommended for optimal integration at the point of load centres because of its numerous benefits^3^. RER DG models especially solar and wind energy are highly recommended because of their environmental sustainability, minimum operational costs and ample accessibility. Unlike conventional fossil fuels, RER DG significantly cut down the greenhouse gas emission, support energy autonomy, and encourage decentralized power generation, which improves the reliability and resilience of the distribution power grid.

A novel short-term interval forecasting technique was proposed in^4^ for estimating the PV power. The meteorological element of the PV station was reconstructed using a second-order extended Markov model (HMM). A trend fusion and fluctuation clustering-based wind speed correction technique was proposed in^5^. First, the input feature was framed using the wind speed trend, and then the numerical weather prediction wind speed error distribution was computed to develop a precise and stable mapping relationship. A novel ultra-short-term wind power forecasting approach was proposed based on the EMD-CC Transformer^6^. The network model is framed, demonstrating the encoder–decoder structure. The historical wind sequence is analyzed in encoder then the wind power prediction is done using the decoder. A frequency voltage active support technique for a hybrid wind farm based on grid-following and grid-forming hierarchical subgroup control was proposed to support the premises for ensuring system stability^7^. A virtual external perturbance-based impedance measurement technique was implemented for grid-connected converter^8^. The proposed technique applied changes to the voltage and current samples at the point of common coupling.

Moreover, the modern energy systems such as microgrids encourage decentralized operation with distrusted power energy resources. Microgrid power networks employ small scale DG units and energy storage systems to operate independently and in synchronize with the main distribution grid. Microgrid with optimized integration of DG units can enhance resilience, reliability and curtail power losses. Moreover, integration of RER based DG unit play an integral part in the transition to smart grid.

A homomorphic encryption-based resilient distributed algorithm was suggested for microgrid energy management to prevent the malevolent data intrusions^9^. A fuel tank dispatch was included as an integral part of the mobile emergency generators for better restoration of power^10^. Authors have suggested a generalized Nash-in-Nash bargaining model for a building for assessing the energy loss and network usage cost in a P2P trading market^11^. The SSA-CNN-BiLSTM-Attention model was proposed to optimize the energy dispatch in the microgrid with enhanced prediction accuracy^12^. An efficient distributed optimization algorithm was developed for solving the dynamic economic dispatch problem in a hybrid microgrid network^13^. The economic dispatch problem was solved for minimizing the total generation expenses.

Energy management (EM) is essential in DG integrated power system networks to monitor the effective utilization of generated power. As the existing traditional power grids are shifting towards smart grid technology, Energy Management System (EMS) play a crucial part in adopting decentralized power generation and energy storage aiding to balance between generation and demand while minimizing greenhouse/carbon emissions and operational costs. EMS incorporates innovative software tools and communication technologies to monitor the operation of DG integrated electrical power networks. They help to forecast energy demand and optimize energy dispatch and make the system operator to initiate informed decision. Real-time control strategy of EM system enhances the efficiency, sustainability and reliability of conventional DPN and microgrid.

Authors have implemented an event-trigger-based resilient distributed energy management system to safeguard the smart grid from false data injection and denial of service attacks^12^. A complete hydrogen energy chain concept was proposed in^14^ to optimize the energy flows in all links of the hydrogen chain-based energy system using a bottom-up long-term investment strategy. A game-theoretic model was developed to examine the manufacturer’s optimal building strategy^15^. The outcome reveals that the manufacturer can build the charging stations whenever the construction cost is fairly low. A multiagent reinforcement learning technique was proposed in^16^ to maximize social welfare with privacy protection in a dynamic energy market. The proposed technique was executed for taking optimal decisions in the P2P energy market. A numerical simulation has been carried out to investigate the heat transfer behavior between the thermoelectric module and the environment^17^.

The optimized DG inclusion in the DPN/microgrid can reduce the total PL, regulate the bus voltages, and enhance the stability and reliability^3^. But the position and size for DG in DPN should be optimized appropriately to achieve utmost assistance from it. Meanwhile, inappropriate inclusions of DGs have led to more PL and unreliable conditions in DPNs^4^.

Researchers have adopted several analytical and meta-heuristic algorithms-based techniques^18–20^ for optimizing the location and ratings of compensation devices such as capacitors, few FACTS devices (SVC, STATCOM and UPFC), and decentralized power generation units. The analytical technique incorporates mathematical computation to solve the optimization problem. The heuristic technique uses randomness to solve optimization problems. Table 1 presents taxonomy of optimization techniques applied for enhancing the performance of RDPN.

**Table 1:** Taxonomy of optimization technique.

Reference	Technique	Objective(s)	Compensation method	Case study test system
^22^	Analytical	RPL minimization	Type I, II, III and IV DG	IEEE 16-bus, 33-bus and 69-bus RDPNs
^23^	Analytical	PL and cost of energy minimizations	Type I and III DG	IEEE 12-bus, 69-bus and 85-bus RDPNs
^24^	ALO	RPL reduction, VP and VSI improvement	Type I and III DG	IEEE 33-bus and 69-bus RDPNs
^25^	BSOA	RPL minimization and VP improvement	Type I and III DG	IEEE 33-bus and 94-bus RDPNs
^26^	Hybrid EHO – PSO	RPL reduction, VP and VSI improvement	Type I DG	IEEE 33-bus, 69-bus and 118-bus RDPNs
^27^	SKHA	RPL minimization	Type I DG	IEEE 33, 69 and 94 bus Portuguese RDPN
^28^	CSA	RPL and operating cost minimizations	Type I, III and IV DGs	Real Egyptian RDPN
^29^	OTCDE	RPL, operating cost and voltage deviation minimizations	Type I and III DG	IEEE 33-bus, 69-bus and 118-bus RDPNs
^30^	DE	RPL and energy loss minimizations	PV, Wind Turbine (WT) and Biogas Generator	IEEE 69-bus RDPN
^31^	SOA	RPL reduction, VP and VSI improvement	PV and WT	IEEE 33, 69, and 85 bus RDPNs
^32^	Hybrid TLBO-GWO	RPL minimization	PV and WT	33 and 69 bus IEEE RDPN
^33^	ISOS	RPL reduction, VP and VSI improvement	Type I and III DG	IEEE 33-bus, 69-bus and 118-bus RDPNs
^34^	CSCA	RPL reduction, VP and VSI improvement	Type I and III DG	IEEE 33-bus and 69-bus RDPNs
^35^	HHO	RPL reduction, VP and VSI improvement	Type I and III DG	IEEE 33-bus and 69-bus RDPNs
^36^	Hybrid Improved GWO-PSO	RPL reduction, VP and VSI improvement	Type I and III DG	IEEE 33-bus and 69-bus RDPNs
^37^	Hybrid LSF- SCA	RPL minimization	Type I, II and III DG	IEEE 33-bus and 69-bus RDPNs
^39^	Hybrid PSO-DE	PL and operating cost minimizations	UPFC	IEEE 30-bus system
^40^	Hybrid SFLA-PSO	Total generation cost, emission, real power transmission losses, and voltage deviation	FACTS devices	IEEE 30-bus system
^41^	hybrid WIPSO-GSA/GWO	RPL minimization and VP improvement	Type I DG and capacitor	33-bus and Indian 85-bus RDPNs
^42^	BFOA	RPL, voltage stability and operating cost minimizations	Type I and III DG	IEEE 33-bus and 69-bus RDPNs
^43^	KHA	RPL, VSI and energy loss minimizations	Type I and III DG	IEEE 33-bus, 69-bus and 118-bus RDPNs
^44^	BA	RPL reduction and VP improvement	Type I and III DG	IEEE 33-bus RDPN
^45^	WOA	PL and voltage deviation reductions	Type I DG and capacitor	IEEE 33-bus RDPN
^46^	AIS	RPL and reactive power loss reduction and VSI enhancement	Type I DG	IEEE 33-bus RDPN

Mathematical expressions based optimization methods^21,22^ were proposed to solve the optimal placement and sizing problem (OPSP) of DG in the radial power distribution network (RPDN). A novel analytical approach was proposed using voltage stability index (VSI) to find the best optimized solution for the OPSP^23^. ALO algorithm was suggested for optimizing the DG location and rating in the different DPNs^24^. BSOA optimization method was implemented to disperse the DG units optimally in the radial DPN. The DG’s location and sizes were optimized to minimize RPL and increase the bus voltages^25^. A hybrid EHO – PSO method was proposed for optimizing the different cases of DG units in IEEE benchmarks radial DPNs. The study considered three IEEE benchmark test systems, viz., 33, 69 and 118-bus RPDNs for validation. The DG locations and ratings were optimized aiming to minimize RPL and line voltage drop and improve distribution line voltage stability^26^. SKHA was suggested to find the solution for the DG OPSP in DPN^27^. The study considered RPL minimization as an objective. The effectiveness of SKHA was verified on the 33 and 69-bus IEEE benchmark RPDNs. CSA method was introduced to optimally integrate three types of DG units into the DPN. The proposed work was tested on a real Egyptian DPN for RPL minimization and operating cost savings^28^. A novel optimization method using OTCDE was proposed to optimize the placement and ratings for multiple units of DGs^29^. Likewise, DE method was proposed to optimize multiple units of DG in the distribution power grid^30^. A metaheuristic technique was proposed using SOA to optimize the DG sites and ratings for PL minimization, bus voltage enrichment and distribution line VS enhancement^31^. Hybrid TLBO-GWO optimization method was proposed to optimize PV and wind turbine systems in the IEEE 33 and 69-bus benchmark RDPNs^32^. The optimal positions and ratings for the DGs were optimized via ISOS algorithm to minimize PL, enhance VP and improve VS of radial DPNs^33^. Multiple units of DGs were optimally integrated into the IEEE 33-bus and 69-bus benchmark DPNs using CSCA method considering a single and multi-objective problems^34^. HHO technique^35^ and GWO-PSO integrated method^36^ were implemented to identify the locations and ratings of different type of DGs. The optimization was done to minimize PL and improve VP of DPN. LSF- SCA hybrid method was suggested to optimize PV and wind turbine (WT) systems to minimize PL and improve the bus voltages of an unbalanced 33-bus benchmark DPN^37^. GA^38^, hybrid PSO-DE^39^ and hybrid SFLA-PSO^40^ methods were proposed to optimize single solar PV system for real PL minimization and bus voltage improvement. The optimal positions and sizes for two units of solar PV systems were optimized using hybrid WIPSO-GSA^41^ and GWO^41^ methods. Likewise, BFOA^42^, KHA^43^, BA^44^, WOA^45^, and AIS^46^ approaches optimized the three units of PV systems locations and capacities in the 33-bus radial DPN for RPL and voltage deviation minimizations.

Analytical methods give inaccurate results and take more time to converge. But, the metaheuristic algorithm methods produced better results than analytical methods and converge in faster rate. However, metaheuristic algorithms also possess their respective limitations and weaknesses. For example, GA, BA, BSOA, ALO, GWO, BFOA, and WOA optimization algorithms are effective in solving small and simple dimensional problems, but become ineffective for large and high dimensional problems. Hence these algorithms often prematurely converge to a local optima solution because of their insufficient diversity in the population and imbalance between the exploitation and exploration. JSA is relatively a novel algorithm compared to the aforementioned algorithm, which is introduced for solving numerous complex and high dimensional problems^47^. JSA effectively solves the nonlinear and complex problems with its diversified search mechanism and balanced exploitation and exploration better results than the analytical methods. Also, the effectiveness of the JSA is addressed for various benchmark functions^47^.

The present study addresses the multi-bjective DG OPSP using JSA. The contribution of the study is highligghted below:

- A multi-objective JSA optimization technique that intelligently maps the food-searching behaviors of jellyfish is implemented to optimize solar PV DG systems for achieving technological benefits, viz., RPL reduction and bus voltage deviation minimization, and VS enhancement.
- The performance of the JSA metaheuristic technique is evaluated on a 33-bus RDPN for (i) single, (ii) two, and (iii) three solar PV DG unit placements.
- Efficacy of the simulation findings is evaluated via a comprehensive comparison study.

The present work is detailed in different sections. Section II details the mathematical formulation of DG placement and sizing problem. Section III presents JSA mathematical modelling and implementation. Section IV explains the simulation outcomes for different cases of PV systems placement and sizing. Section V concludes the significant findings of the present work.

## Problem formulation

**Fig. 1:**
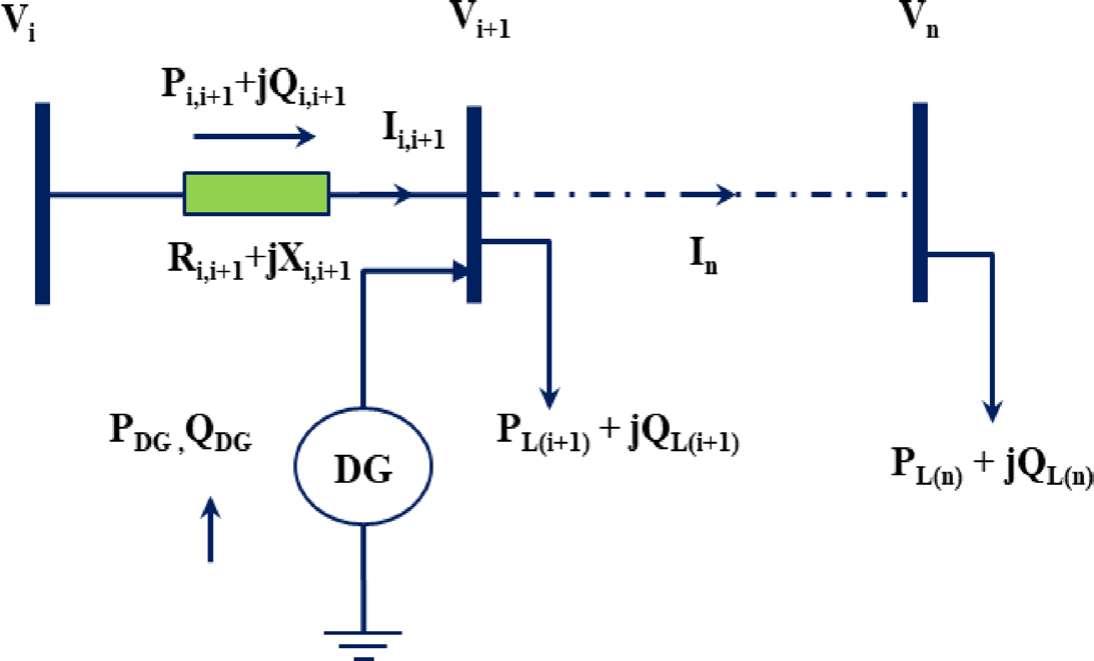
Radial PDN with DG placement.

The identification of critical buses and ratings for DG units in the RDPN is a complex and non-linear problem. This study aims to optimize single and multiple units of PV systems as DGs to minimize RPL, minimize VD, and enhance line VS. Single line diagram (SLD) of a typical RDPN having ‘n’ number of buses and ‘k” number of distribution lines is shown in Fig. 1. The distribution lines are represented by equivalent series impedances. The power flow in the RDPN is expressed as simplified recursive equations.

Equations (1), (2), (3) give the expression for the real and reactive power flow, and the bus voltage^48^.

For a bus, i = 1, 2, …., n and k = 1, 2,….n-1.

1 $$P_{i + 1} = \left[ {P_{k} - \left( {R_{k} \frac{{P_{k}^{2} + Q_{k}^{2} }}{{\left| {V_{i} } \right|^{2} }}} \right) - P_{L(i + 1)} + P_{DG}^{{}} } \right]$$

2 $$Q_{i + 1} = \left[ {Q_{k} - \left( {X_{k} \frac{{P_{k}^{2} + Q_{k}^{2} }}{{\left| {V_{i} } \right|^{2} }}} \right) - Q_{L(i + 1)} + Q_{DG}^{{}} } \right]$$

3 $$\left| {V_{i + 1} } \right|^{2} = \left[ {\left| {V_{i}^{2} } \right| + \frac{{R_{k}^{2} + X_{k}^{2} }}{{\left| {V_{i} } \right|^{2} }}\left( {P_{k}^{2} + Q_{k}^{2} } \right) - 2\left( {R_{k} P_{k} + X_{k} Q_{k} } \right)} \right]$$

The branch current is expressed in Eq. (4).

4 $$I_{k} = \sqrt {\frac{{P_{k}^{2} + Q_{k}^{2} }}{{\left| {V_{i} } \right|^{2} }}}$$

Whenever the current passes through a distribution line, power losses occur. Equations (5) and (6) express the real (P_loss_) and reactive (Q_loss_) power loss in a branch, respectively^48^.

5 $$P_{loss (k)} = \left| {I_{k} } \right|^{2} R_{k}$$

6 $$Q_{loss (k)} = \left| {I_{k} } \right|^{2} X_{k}$$

Total P_loss_ and Q_loss_ of ‘n’ bus and ‘nb’ branch RDPN are expressed in Eqs. (7),  (8), respectively^48^.

7 $$P_{Tloss} = \left[ {\sum\limits_{k = 1}^{n - 1} {P{}_{loss (k)}} } \right]$$

8 $$Q_{Tloss} = \left[ {\sum\limits_{k = 1}^{n - 1} {Q{}_{loss (k)}} } \right]$$

Usually P_Tloss_ more dominant than Q_Tloss_ since line resistances are higher than reactance in DPN. Hence, the present work only considers RPL minimization as the prime objective.

RPL along a branch of the RDPN after the optimal inclusion of DG units is mathematically expressed in Eq. (9).

9 $$P_{loss(k)}^{DG} = \left[ {R_{k} \frac{{\left( {P_{k} - P_{DG(i + 1)} } \right)^{2} + \left( {Q_{k} - Q_{DG(i + 1)} } \right)^{2} }}{{\left| {V_{i} } \right|^{2} }}} \right]$$

P_Tloss_^DG^ after the optimal inclusion of DGs is expressed in Eq. (10).

10 $$P_{Tloss}^{DG} = \left[ {\sum\limits_{k = 1}^{n - 1} {P_{loss(k)}^{DG} } } \right]$$

### Objective function

The multiple objectives considered in this study are framed as a single objective function using weighted sum method (WSM) as expressed in Eq. (11).

11 $$MOF = \min \left( {\delta_{1} f_{1} + \delta_{2} f_{2} + \delta_{3} f_{3} } \right)$$

Where, δ_1_, δ_2_, and δ_3_ are the weightage factors. The values for the weightage factors must be appropriately selected such that their sum must be unity.

#### RPL minimization (f_1_)

Total RPL (P_Tloss_) is the prime objective of the DG optimization study. Power loss minimization is achieved via measurement of power loss index (PLI)^49^. The mathematical expression for PLI is given in Eq. (12). Minimum PLI results in maximum PL reduction.

12 $$PLI = \left( {\frac{{P_{Tloss}^{DG} }}{{P_{Tloss} }}} \right)$$

#### Bus voltage deviation minimization (f_2_)

Bus VD minimization is the second of the three objectives. Bus VD cab be reduced via minimization of bus voltage deviation index (VDI)^49^ given in Eq. (13). Reduction of bus VDI increases bus voltage.

For i = 1, 2 ……n

13 $$VDI_{i} = \left| {V_{1} - V_{i} } \right|$$

#### Voltage stability maximization (f_3_)

Voltage stability enhancement is the third and last objective of the DG optimization study. The present work assesses the voltage stability of the DPN via VSI measurement^42^. Equation (14) gives the mathematical expression for the VSI.

For i = 1, 2… n and k = 1,2,….n-1

14 $$VSI_{i} = \left\{ {\left| {V_{i} } \right|^{4} } \right\} - 4\left\{ {P_{k} X_{k} - Q_{k} R_{k} } \right\}^{2} - 4\left\{ {P_{k} R_{k} + Q_{k} X_{k} } \right\}\left| {V_{i} } \right|^{2}$$

A stable DPN is the one with VSI values closer to unity at all the buses. If the VSI of any one of the buses reads zero or approaches near zero, then the DPN is considered to be an unstable system. VSI is a scalar value and is computed from load flow results data. The VSI maximization is obtained by minimizing the reciprocal value of Eq. (14).

### Operational constraints

The optimal solution to a DG placement problem should not violate any operational constraints of DPN. Equations (15)–(18) give the real power balance, bus voltage, thermal capacity, and DG power rating constraints considered in the present DG optimization study^42^.

15 $$P_{S} + \sum\limits_{i = 1}^{{N_{DG} }} {P_{DG} (i) = \sum\limits_{k = 1}^{n - 1} {P_{loss} (j)} } + \sum\limits_{j = 1}^{n} {P_{L} (j)}$$

16 $$V_{\min } \le V_{i} \le V_{\max }$$

17 $$\left| {I_{k} } \right| \le \left| {I_{k,\max } } \right|$$

18 $$P_{\min }^{DG} \le P_{T}^{DG} \le P_{\max }^{DG}$$

Where,

19 $$P_{\min }^{DG} \le 0.1\sum\limits_{i = 2}^{n} {P(i)}$$

20 $$P_{\max }^{DG} \le 0.8\sum\limits_{i = 2}^{n} {P(i)}$$

### DG modeling: PV system

The present study analyzes the impact of PV system penetration for achieving technological benefits in DPN. The PV system is modelled as a P-type DG unit and injects only real power into the RRDN. Equation (21) presents the mathematical model for a PV system^49^.

21 $$P_{pv} = \left\{ {\begin{array}{*{20}c} {P_{pvr} \times \left( {\frac{G}{{G_{r} }}} \right),0 \le G \le G_{r} } \\ {P_{pvr,} G_{r} \le G} \\ \end{array} } \right\}$$

Since the proposed DG placement and sizing problem is solved from planning perpective, the solar radiation data for PV DG system is neglected.

## JSA metaheuristic technique: modeling and implementation

JSA is a novel metaheuristic algorithm introduced^47^ to solve numerous optimization problems. JSA addresses complex optimization problems effectively through its diverse search ability, an adaptive search approach, and a balanced exploration-exploitation process. These unique features greatly evade local optima entrapment and fast convergence. JSA mimics the food search behaviors of jellyfish. Jellyfish adopt two types of search mechanisms, viz., diversification and intensification, to capture nutrition like fish eggs, larvae, etc., in the ocean current and jellyfish swarm. JSA switches between search mechanisms via a time control mechanism (TCM). The mathematical background of JSA implementation is presented below.

### Population initialization

JSA adopts a unique tactic known as a chaotic map for initializing the population. Equation (22) expresses the population initialization.

22 $$X_{i + 1} = \eta X_{i} \left( {1 - Y_{i} } \right)\begin{array}{*{20}c} , & {0 \le X_{i} \le 1} \\ \end{array}$$

And

23 $$X_{0} \in \left( {0,1} \right)\begin{array}{*{20}c} , & {X_{0} \notin \left\{ {0,0.25,0.75, 0.5,1.0} \right\}} \\ \end{array}$$

### Ocean current (OC) search movement

The OC has rich quantities of nutrients. Hence, jellyfish follow the ocean current (OC) in search of nutrition. The direction of OC is discovered using Eq. (24).

24 $$\overrightarrow {trend} = X^{*} - \beta \times rand\left( {0,1} \right) \times \mu$$

Where, ‘X*’ is the best jellyfish position in the search space.

The jellyfish upgrades its location via Eqs. (25),  (26).

25 $$X_{i} (t + 1) = X_{i} (t) + rand(0,1) \times \overrightarrow {trend}$$

26 $$X_{i} (t + 1) = X_{i} (t) + rand(0,1) \times X^{*} - \beta \times rand(0,1) \times \mu$$

### Jellyfish search movement

Jellyfish swarm movement is represented as type ‘A’ and type ‘B’ motions. Type ‘A’ and type ‘b’ movements are characterized as passive and active motion, respectively. In the beginning of the optimization process, jellyfish swarms tend to follow type ‘A’ motion. But, later it follows type ‘B’ motion. Equation (27) gives the jellyfish movement following a type ‘A’ motion.

27 $$X_{i} (t + 1) = X_{i} (t) + \gamma \times rand(0,1) \times \left[ {H_{b} - L_{b} } \right]$$

The jellyfish direction is explored in type ‘B” motion via considering a jellyfish, ‘j,’ alongside the one chosen (jellyfish, ‘i’) in a random process. If the nutrition around the jellyfish, ‘j,’ is more compared to the location of jellyfish, ‘i,’ then jellyfish, ‘i,’ directs towards jellyfish, ‘j.’ Otherwise, jellyfish, ‘i’ directs away from jellyfish, ‘j.’ Similarly, remaining jellyfish inside the swarm move and occupy the best position to consume the food. The mathematical illustration for the jellyfish movement and its updated position is given in Eqs. (28)–(30).

28 $$\overrightarrow {Step} = rand(0,1) \times \overrightarrow {Direction}$$

29 $$\overrightarrow {Direction} = \left\{ {\begin{array}{*{20}c} {\begin{array}{*{20}c} {X_{j} (t) - X_{i} (t)} & {if} & {f(X_{i} ) \ge f(X_{j} )} \\ \end{array} } \\ {\begin{array}{*{20}c} {X_{i} (t) - X_{j} (t)} & {if} & {f(X_{i} ) < f(X_{j} )} \\ \end{array} } \\ \end{array} } \right.$$

30 $$X_{i} (t + 1) = X_{j} (t) + \overrightarrow {Step}$$

Where, f (X) refers to a fitness function for the location ‘X’.

### Time control mechanism (TCM)

OC embraces a bulk quantity of nutritious food. Therefore, the jellyfish creates a swarm to search for food in the OC. The OC changes its direction for a temperature or wind direction change. Under this circumstance, the jellyfish crafts another swarm and directs its movement towards the OC. However, JSA includes TCM to normalize the jellyfish movement inside and outside the swarm. A time control function (TCF) and constant ‘c_0_’ is introduced in the TCM to normalize the jellyfish movement. Equation (31) mathematically expresses TCF.

31 $$c(t) = TCF = \left| {\left( {1 - \frac{t}{{Iter_{\max } }}} \right) \times (2 \times rand(0,1) - 1)} \right|$$

Where, (1-c(t)) signifies the movement of jellyfish inside a swarm. For ‘rand (0, 1)’ greater than (1-c(t)), the jellyfish follows type ‘A’ motion; otherwise jellyfish follows type ‘B’ motion.

### Boundary conditions

The jellyfish circulates randomly inside an ocean. Hence, its position must be regularized within a specified boundary condition whenever it goes beyond the search area to have a better solution. Equation (32) illustrates the boundary condition normalization.

32 $$X_{i,d}{\prime} = \left\{ {\begin{array}{*{20}c} {\begin{array}{*{20}c} {\left( {X_{i,d} - H_{b,d} } \right) + L_{b,d} } & {if} & {X_{i,d} > H_{b,d} } \\ \end{array} } \\ {\begin{array}{*{20}c} {\left( {X_{i,d} - L_{b,d} } \right) + H_{b,d} } & {if} & {X_{i,d} < L_{b,d} } \\ \end{array} } \\ \end{array} } \right.$$

**Fig. 2:**
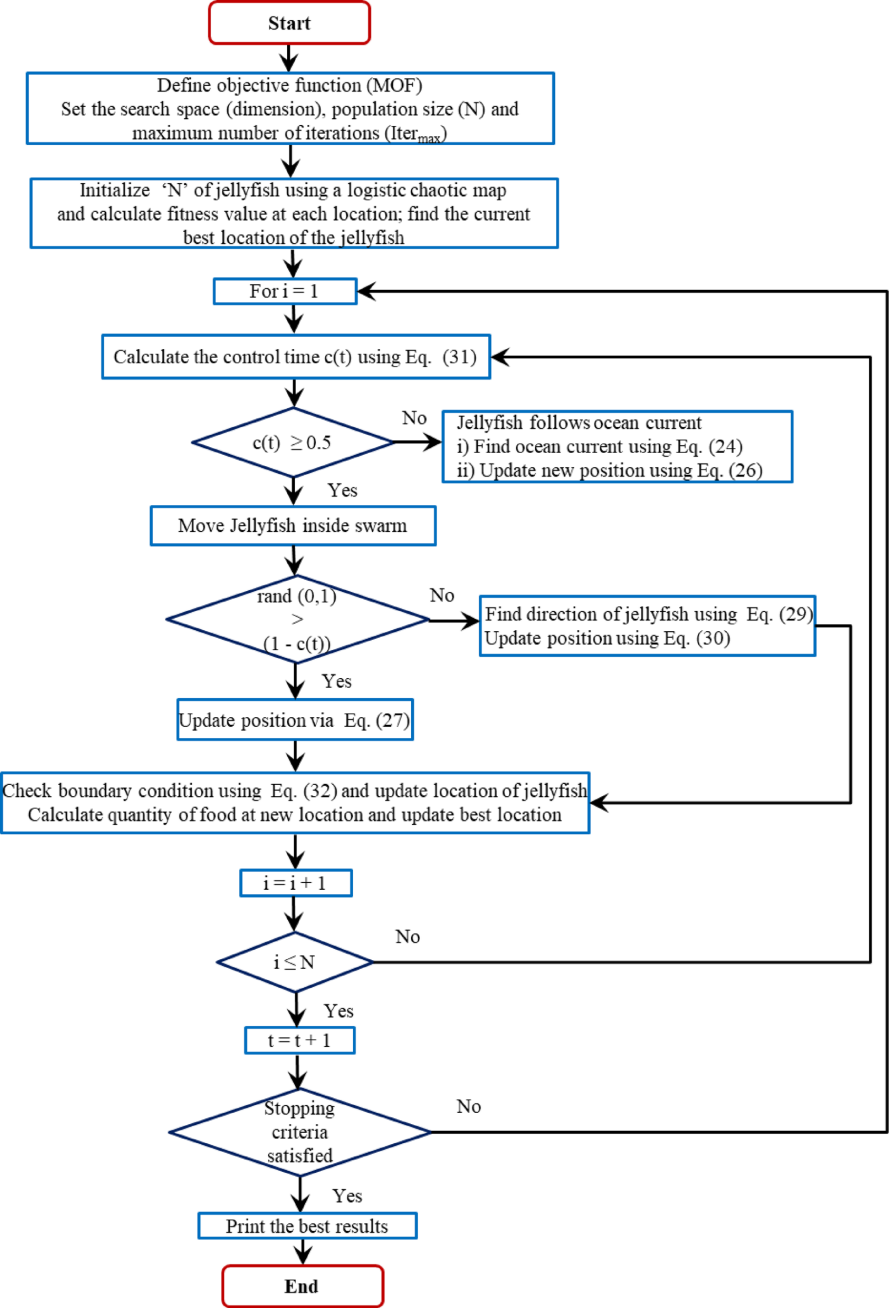
JSA flowchart.

Where, X_i, d_ and X^’^_i, d_ denote jellyfish’s actual position and updated position, respectively. The JSA algorithm is presented graphically as a flowchart in Fig. 2.

The JSA application for the DG placement and sizing problem is presented as algorithms below.

Step 1) Input the necessary data for the 33-bus IEEE radial DPN.
Step 2) Calculate P_Tloss_, VDI, and VSI for the radial DPN without DG placement.
Step 3) Initialize population size, iteration, and control parameters of JSA.
Step 4) Find the initial candidate solutions using Eq. (22) and map the solution with jellyfish location.
Step 5) Compute the fitness value for every candidate solution.
Step 6) Fix iteration to 1.
Step 7) Update the jellyfish location using Eqs. (25), (26).
Step 8) Determine the fitness value for the updated jellyfish location.
Step 9) Compare the fitness values of present and previous locations of jellyfish. Assign the solution that gives the minimum fitness value as the elite solution.
Step 10) Check for constraint violation and iteration number. If the iteration count is less than the maximum value, increase the iteration by 1 and go to Step 7.
Step 11) Print the optimized DG positions and ratings.

## Simulation outcomes and discussion

The proposed JSA approach for DG optimization problem is executed in MATLAB 2022b software. The simulink examination was performed on Intel i3 core processor featured personal computer. The input variables of JSA and boundary conditions for the decision variables are listed in Table 2. The optimal solution for the multi-objective function (MoF) is solved using the WSA. The fitness value for MoF is examined for different probabilities of weights as shown in Table 2. The combination weights that give the least fitness value for MoF are chosen as the feasible weights. For the present study, δ_1_, δ_2_, and δ_3_ values are approximated as 0.6, 0.3, and 0.1, respectively. Table 3 gives the best fitness values obtained for different combinations of δ_1_, δ_2_, and δ_3_.

**Table 2:** JSA parameter and decision variables.

Variable	Values
No. of populations	30
No. of iterations	100
MVA_base_	100
kV_base_	12.66
Minimum V_bus_ constraint	0.95p.u
Maximum V_bus_ constraint	1.05p.u
$P_{{\min }}^{{DG}}$	60 kW
$P_{{\max }}^{{DG}}$	3000 kW

**Table 3:** Selection of weights for δ_1_, δ_2_ and δ_3_.

δ_1_	δ_2_	δ_3_	Fitness value
0.5	0.1	0.4	0.6667
0.5	0.2	0.3	0.6229
0.5	0.3	0.2	0.5683
0.5	0.4	0.1	0.5163
0.4	0.2	0.4	0.6534
0.4	0.3	0.3	0.5980
0.4	0.4	0.2	0.5453
0.4	0.5	0.1	0.5087
**0.6**	**0.3**	**0.1**	**0.5034**
0.6	0.2	0.2	0.5672
0.7	0.2	0.1	0.6201
0.7	0.1	0.2	0.5317
0.8	0.1	0.1	0.5856

Significant values are in bold.

**Fig. 3:**
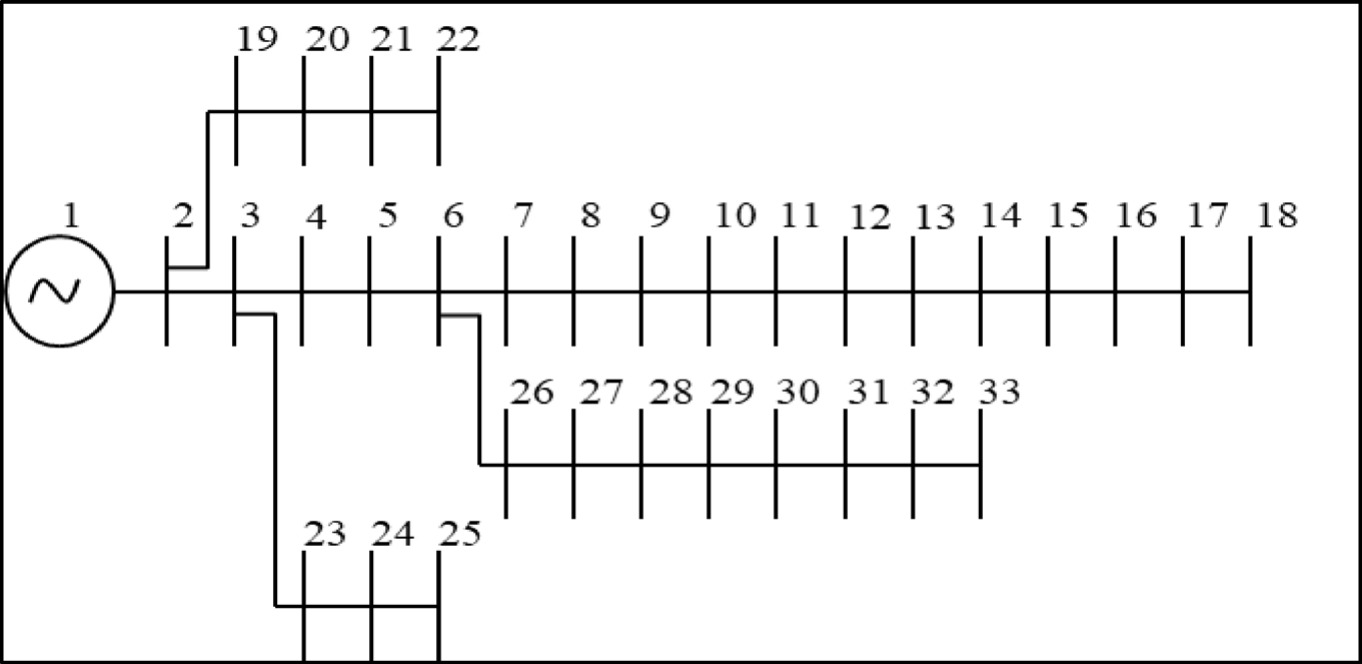
33-bus radial DPN SLD.

Figure 3 shows the SLD of IEEE 33-bus radial DPN considered in this study^50^. The simulation findings are investigated for three cases of solar PV-DG placements. They are: (i) single DG, (ii) two DGs, and (iii) three DGs. The impact of different cases of PV DG optimization are analyzed in terms of total RPL, bus voltage deviation, bus voltages, and line voltage stability. The real-time solar uncertainty is ignored in all cases of PV DG allocation. Further, a constant solar radiation is assumed.

### IEEE 33-bus radial DPN: overview

The test system carries 33 numbers of buses and 32 numbers of branches. The balanced 33-bus radial DPN supplies 3.72 MW - P_L_ and 2.3 MVAr – Q_L_^42^.

### Simulation findings without DG placement

**Fig. 4:**
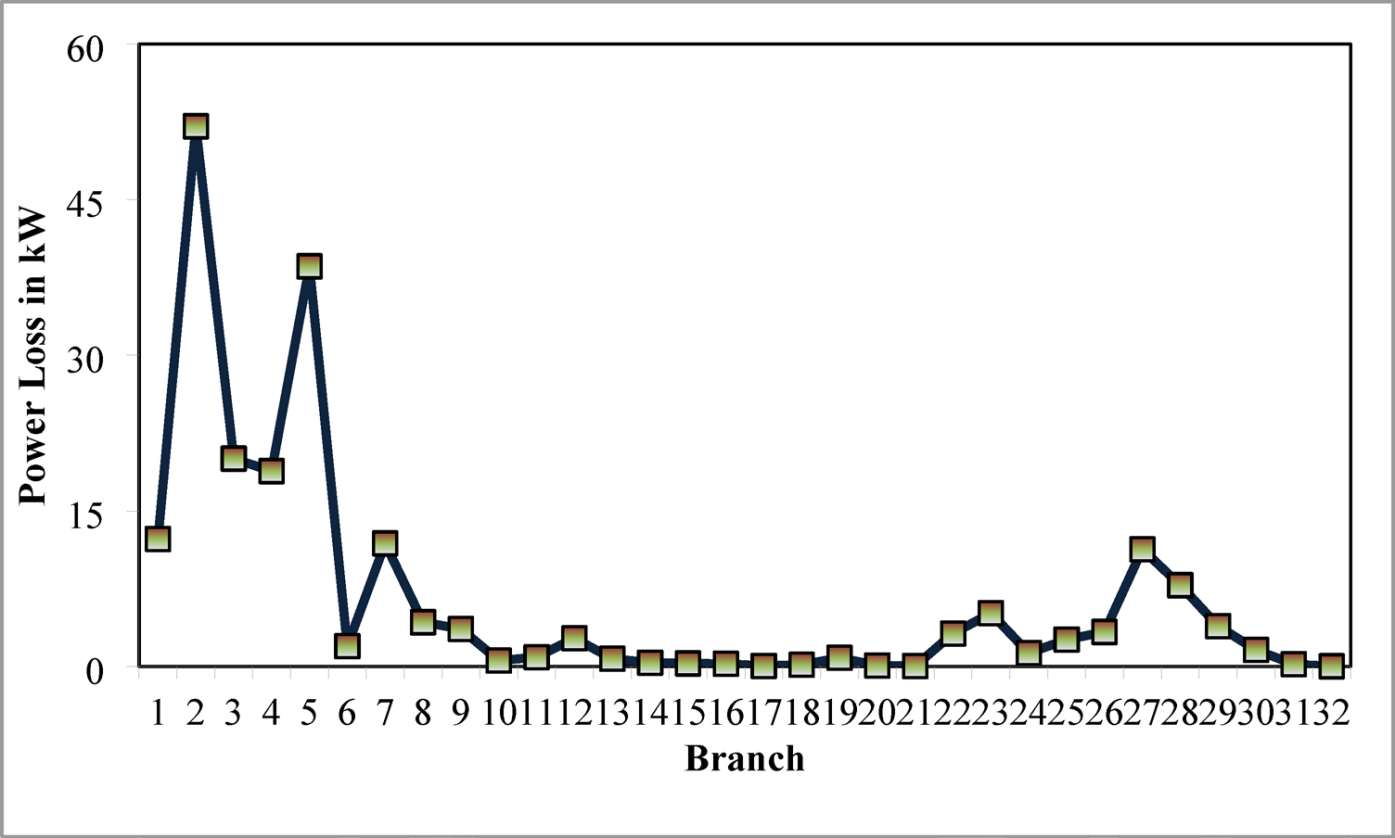
RPL of 33-bus radial DPN without DG.

**Fig. 5:**
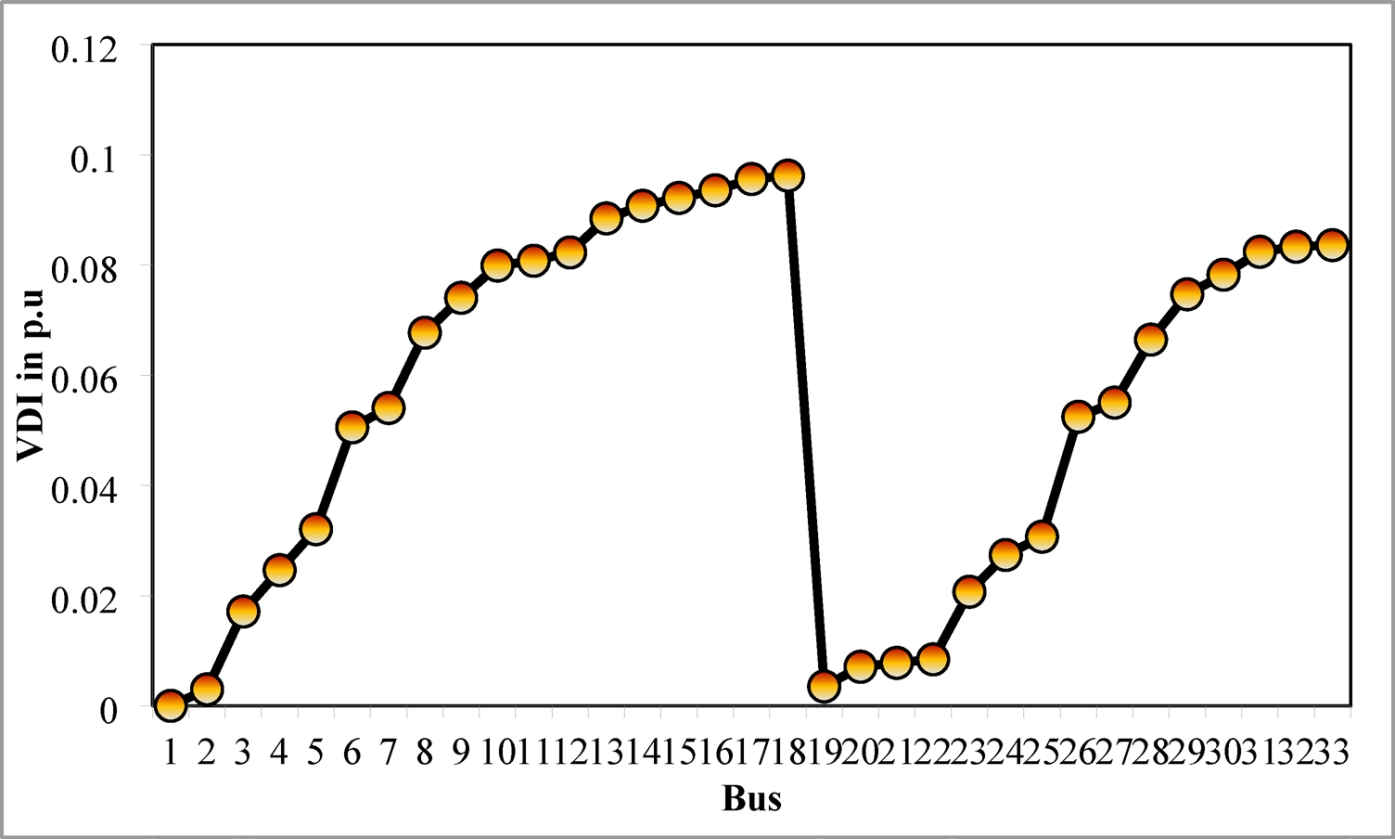
VDI without DG placement.

**Fig. 6:**
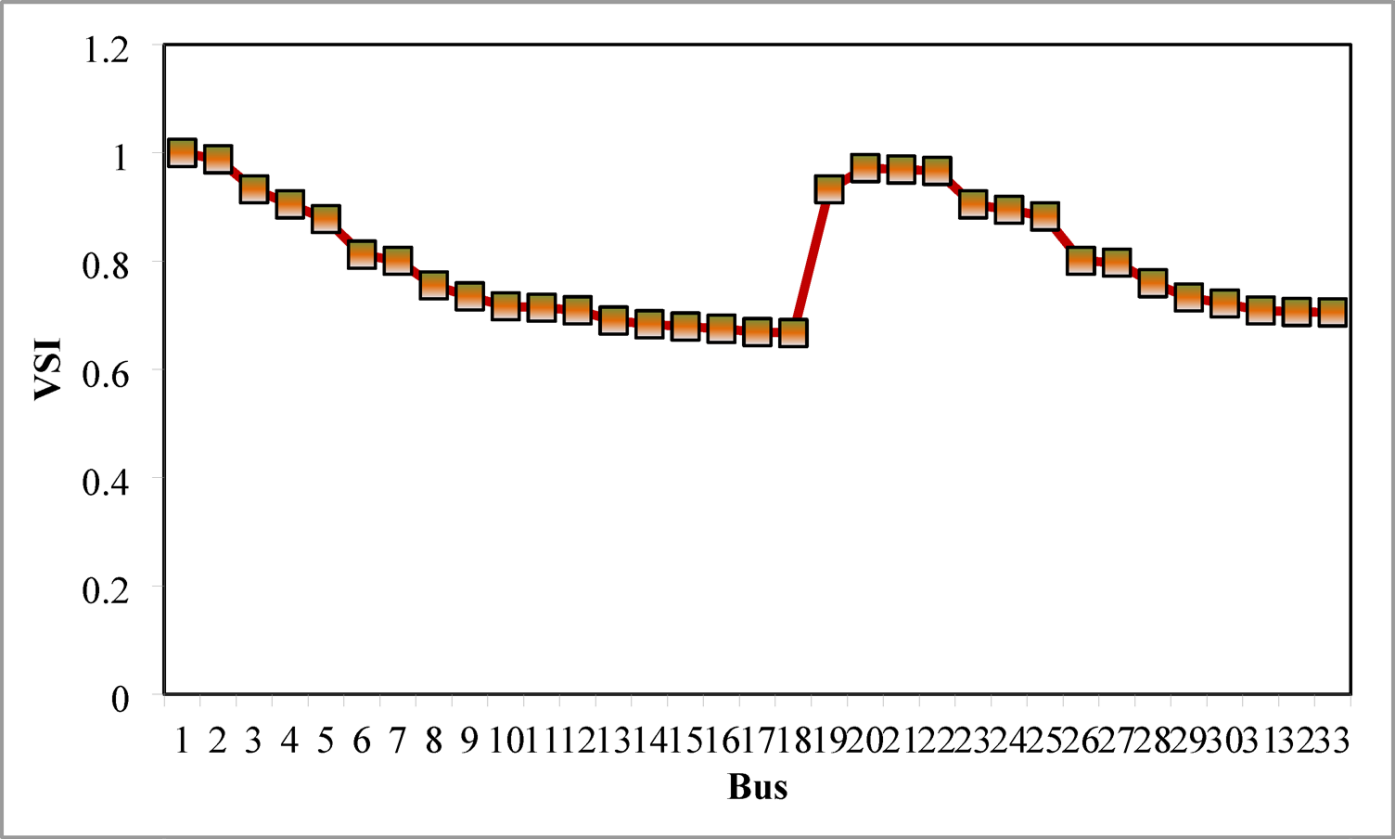
VSI without DG placement.

**Fig. 7:**
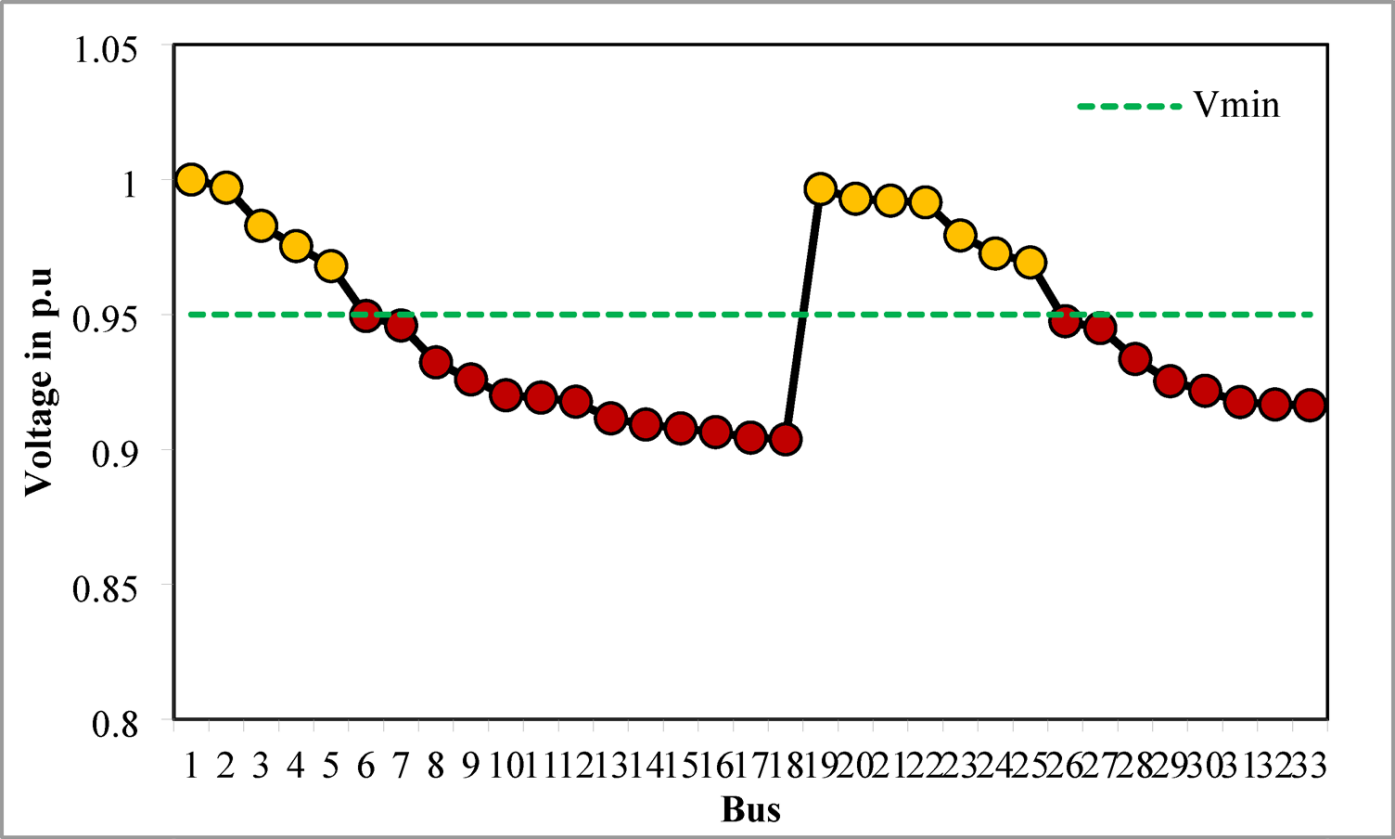
V_bus_ without DG placement.

The simulation findings of 33-bus RPDN without DG placement are essential for measuring the significance of optimized simulation findings. The simulation findings for without DG placement are obtained via backward-forward sweep (BFS) algorithm power flow execution^48^ and are referenced as the base case. For a base case condition, total RPL, minimum bus voltage (V_min_), and minimum voltage stability index (VSI_min_) are 210.98 kW, 0.9038 per unit (p.u.), and 0.6671, respectively. Figures 4, 5, 6 present base case simulation results for RPL, bus VDI, and line VSI, respectively. Figure 7 shows the bus voltages of the 33-bus RDPN without PV-DG placement. The bus voltages are presented in per unit (p.u.) on the 100 MVA_base_ and 12.66 kV_base_. It is noticed from Fig. 5 that far-end-located buses experience more voltage deviation (VD) than the buses near the substation. The total VD of the test system is 1.8047 p.u. and the maximum VDI is 0.0962 p.u. Furthermore, 63.63% of buses (21 out of 33) have voltages below the required level (0.95 p.u.).

Table 4 presents the simulation findings for the optimized inclusion of a single PV system.

**Table 4:** Optimal solution for a single PV DG placement.

Parameter	Simulation findings	
	Without DG	With one DG
Real PL in kW	210.98	102.89
VDI_total_ in p.u	1.8047	0.5331
VDI_min_ in p.u	0.0962	0.0393
V_min_ in p.u	0.9038	0.9607
VSI_min_	0.6671	0.7559
Optimal position(s)	−	6
Optimal size(s) in kW	−	2334.6
Iterations (no.)	−	6
Convergence time (secs)	−	24.4

### Simulation findings with one PV DG placement

**Fig. 8:**
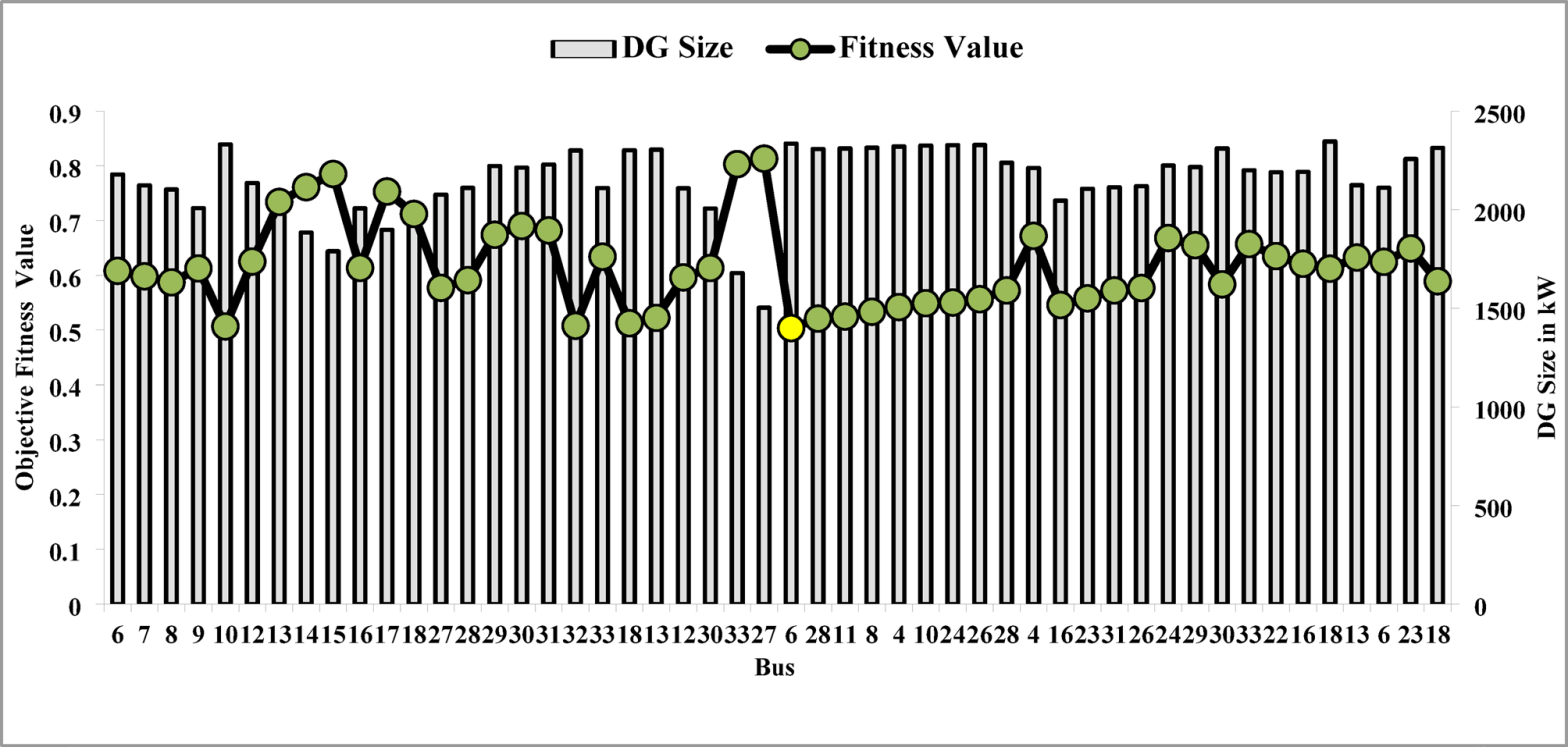
Optimal bus selection for single DG.

**Fig. 9:**
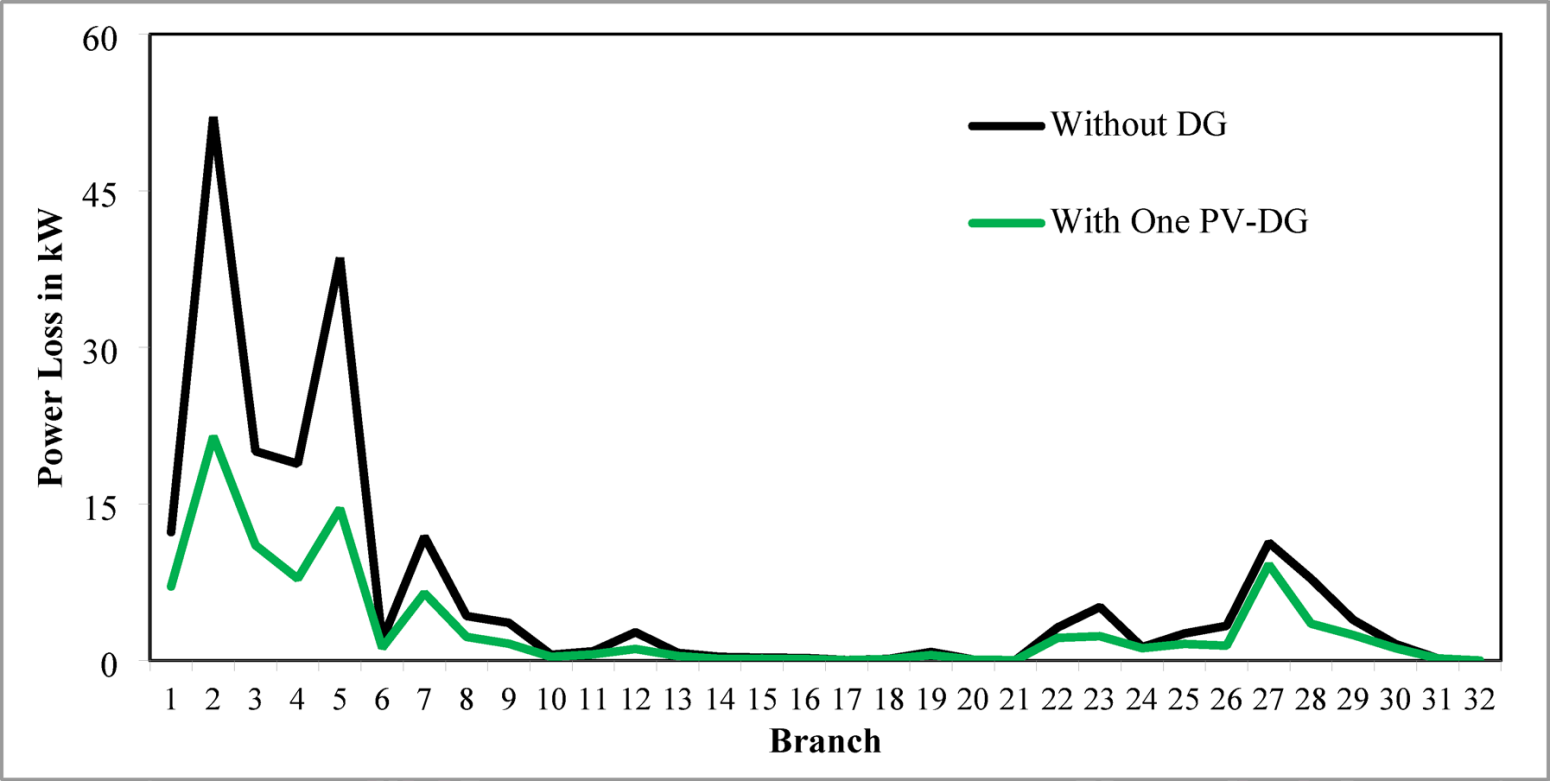
33-bus radial DPN RPL after single DG placement.

**Fig. 10:**
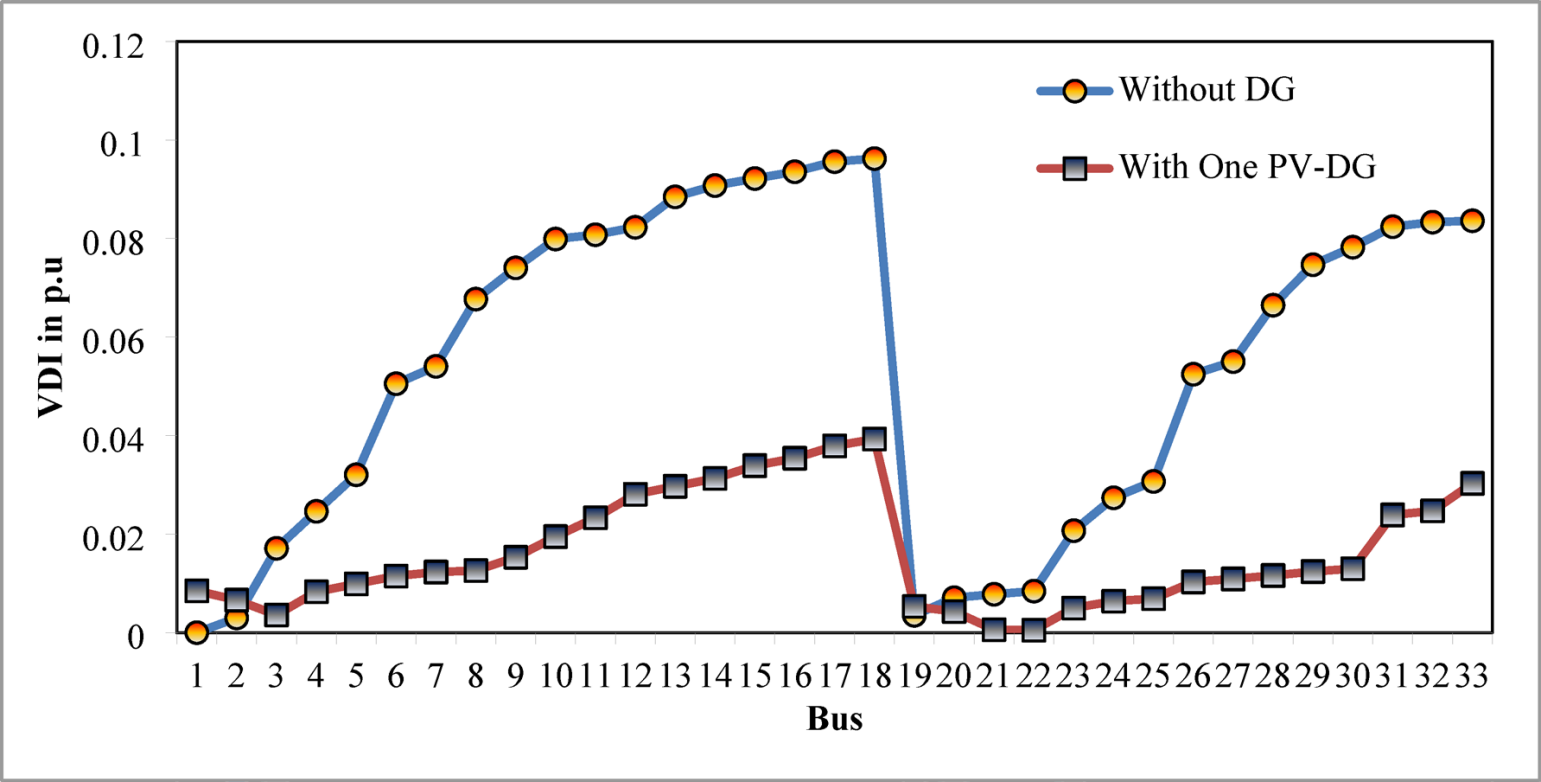
33-bus radial DPN VDI after single DG placement.

**Fig. 11:**
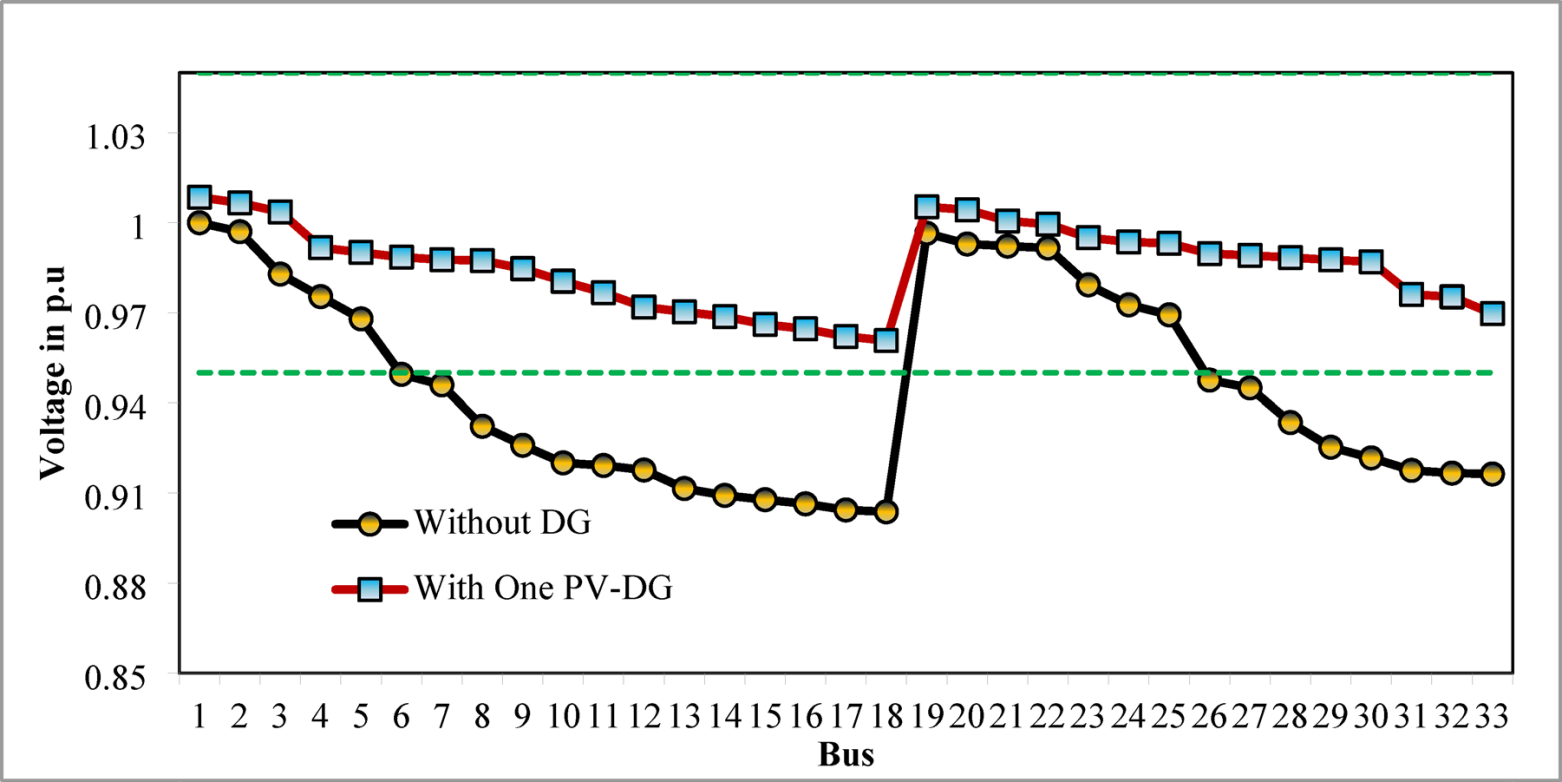
V_bus_ of 33-bus radial DPN after single PV DG placement.

**Fig. 12:**
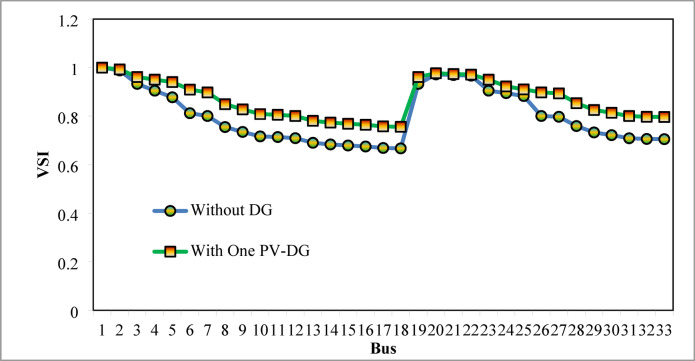
33-bus radial DPN VSI after single DG placement.

**Fig. 13:**
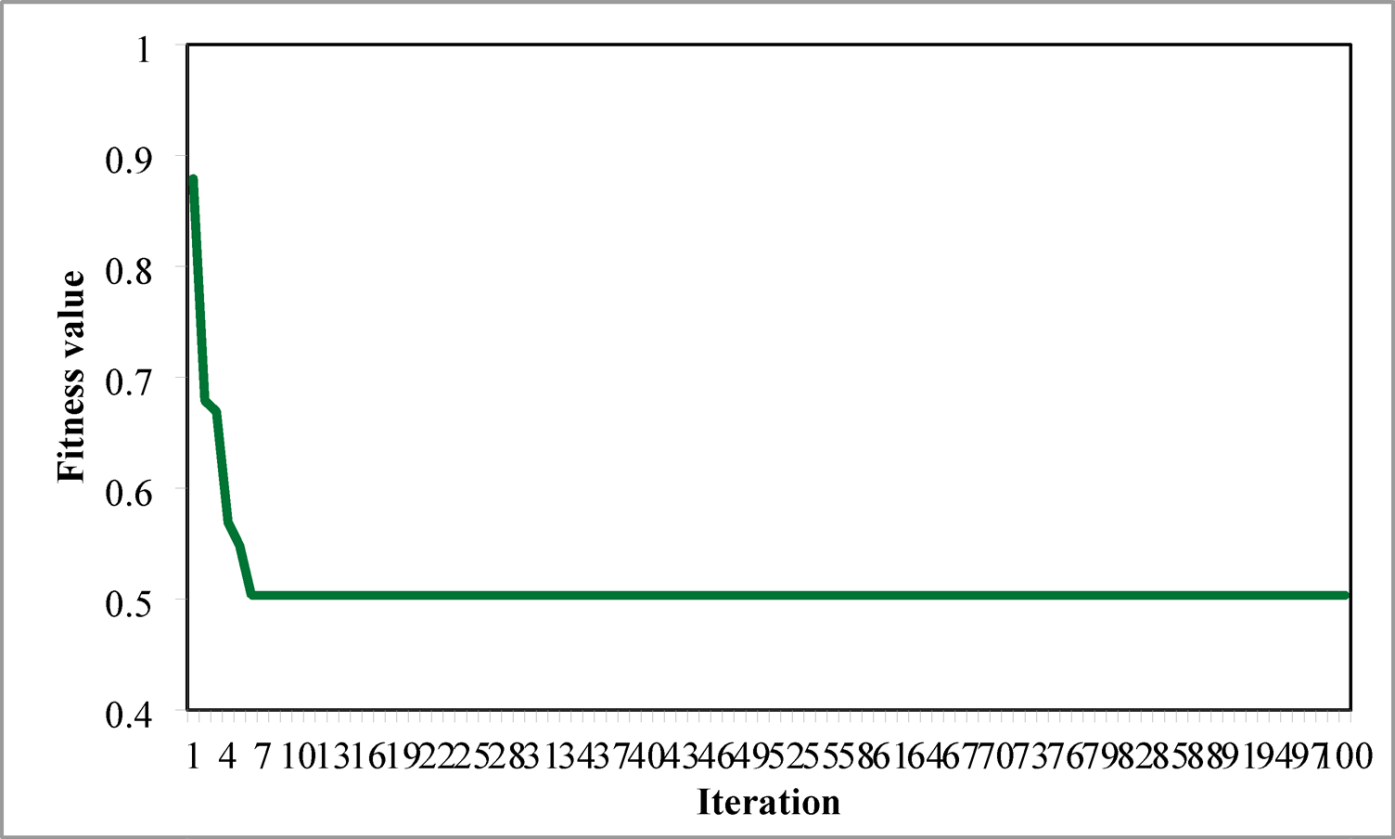
Convergence curve of JSA for a single DG placement.

The optimal solution is obtained in 6 iterations and 24.4 s. Figure 8 illustrates the optimal solution for DG location, size, and objective function fitness value in each run of 50 independent runs. JSA optimizes the single PV DG in the 6th bus of the test system with a 2334.6 kW rating. JSA optimized single PV DG placement gives best solution in the 26th simulation run. The same has been highlighted in the illustration of Fig. 8. The total RPL is minimized from 210.98 kW to 102.89 kW and VDI_total_ is cut down to 0.533 p.u. after the optimized DG inclusion. Furthermore, the VDI_min_, V_min,_ and VSI_min_ are improved to 0.0393 p.u., 0.9607 p.u., and 0.7559, respectively. Figure 9 presents the RPL variation before and after the DG inclusion. Figures 10, 11,12 showcase the optimized solution of VDI, bus voltage, and VSI, respectively, for a single DG allocation. The test system has seen a significant voltage profile enhancement in all buses following the DG placement. Moreover, none of the buses of the test system records a voltage magnitude below the specified V_min_ (0.95 p.u.). Figure 13 presents the convergence curve of JSA for a single DG placement.

### Simulation findings with two PV DGs placement

**Table 5:** Optimal solution for two PV DGs placement.

Parameter	Simulation findings	
	Without DG	With two DGs
Real PL in kW	210.98	82.99
VDI_total_ in p.u	1.8047	0.6518
VDI_min_ in p.u	0.0962	0.0301
V_min_ in p.u	0.9038	0.9698
VSI_min_	0.6671	0.8848
Optimal position(s)	−	13 30
Optimal size(s) in kW	−	822.8 1089.5
Iterations (no.)	−	8
Convergence time (secs)	−	29.4

**Fig. 14:**
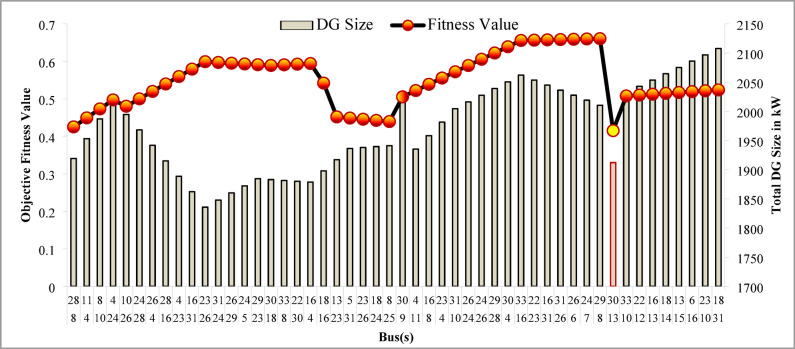
Optimal bus selection for two DGs.

**Fig. 15:**
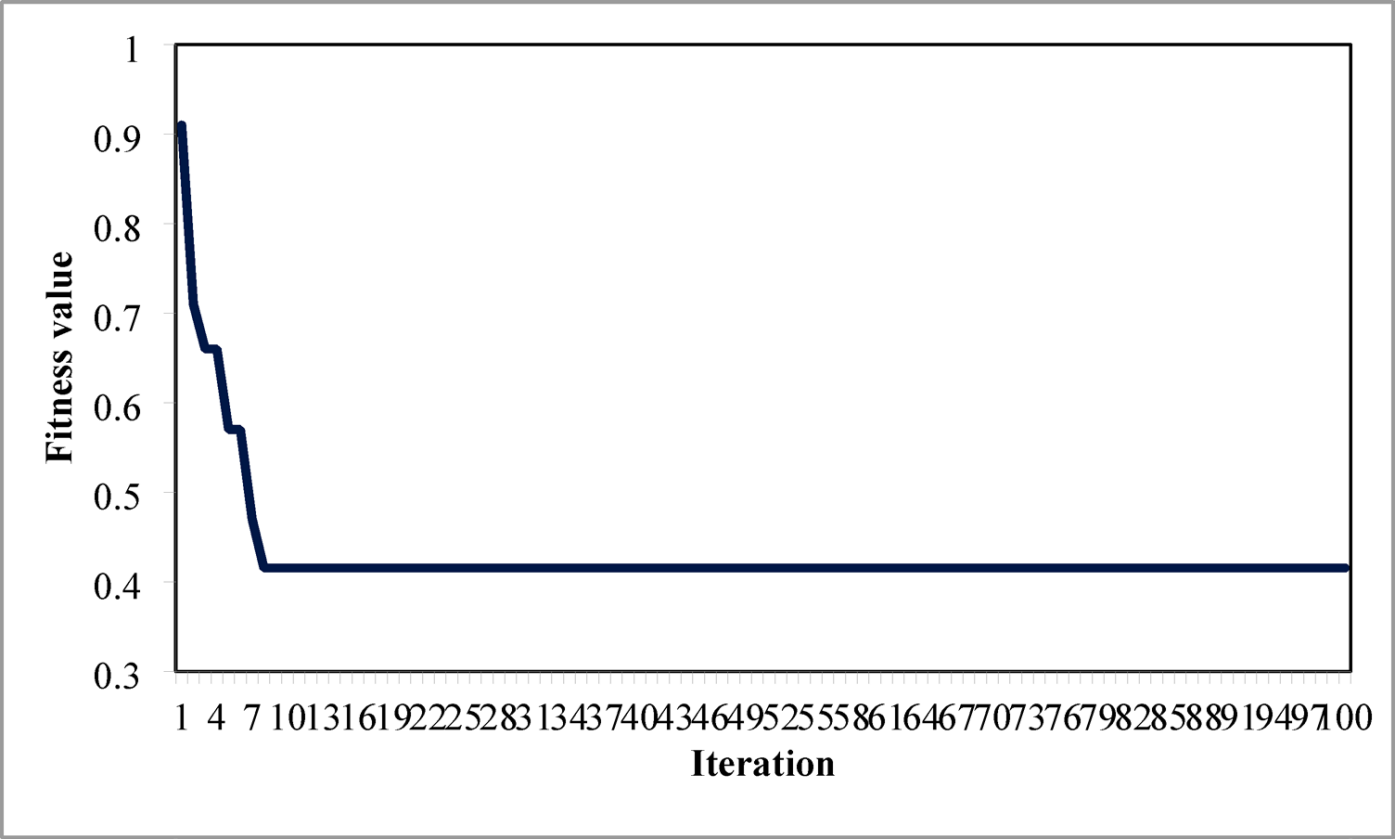
Convergence curve of JSA for a two DG placements.

**Fig. 16:**
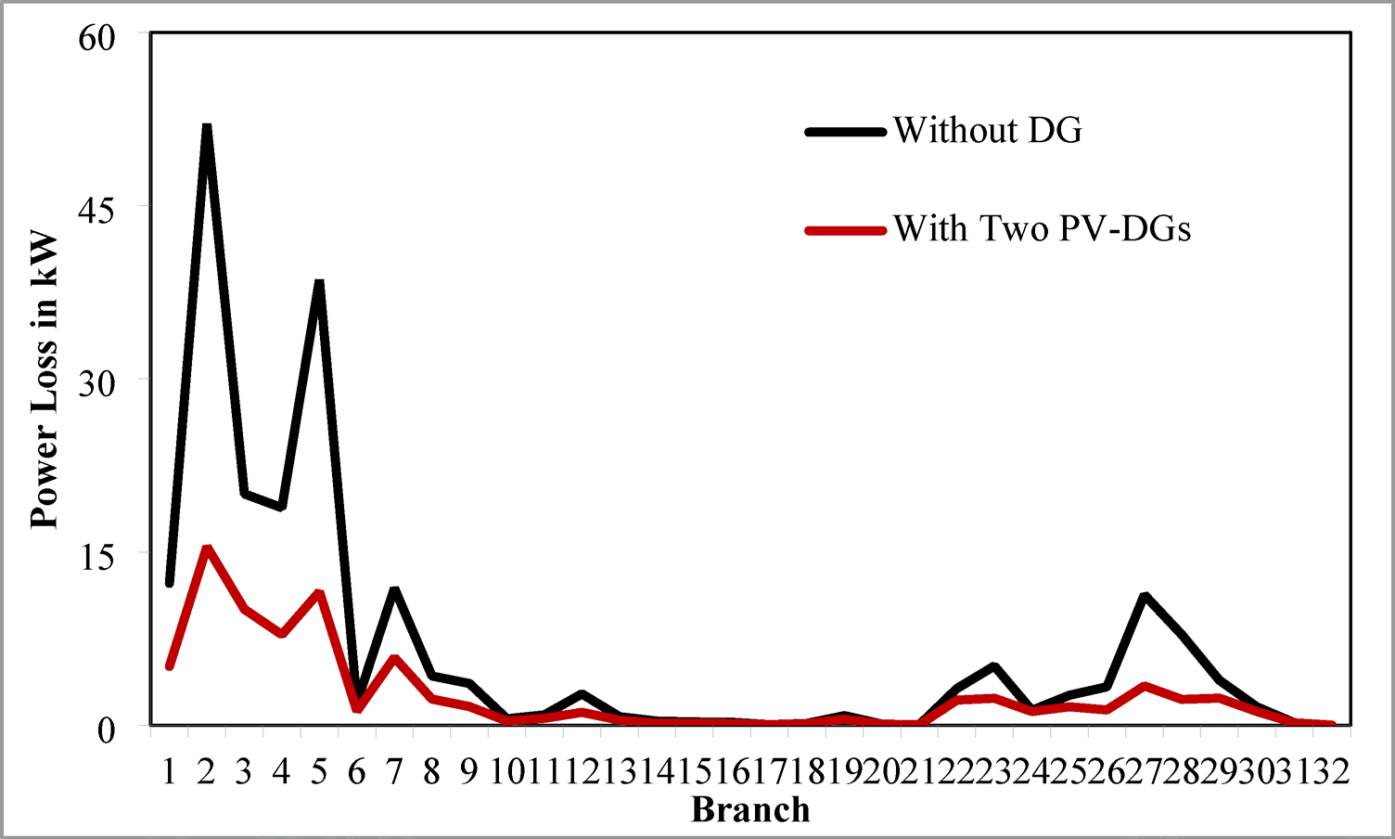
33-bus radial DPN RPL after two DG placements.

**Fig. 17:**
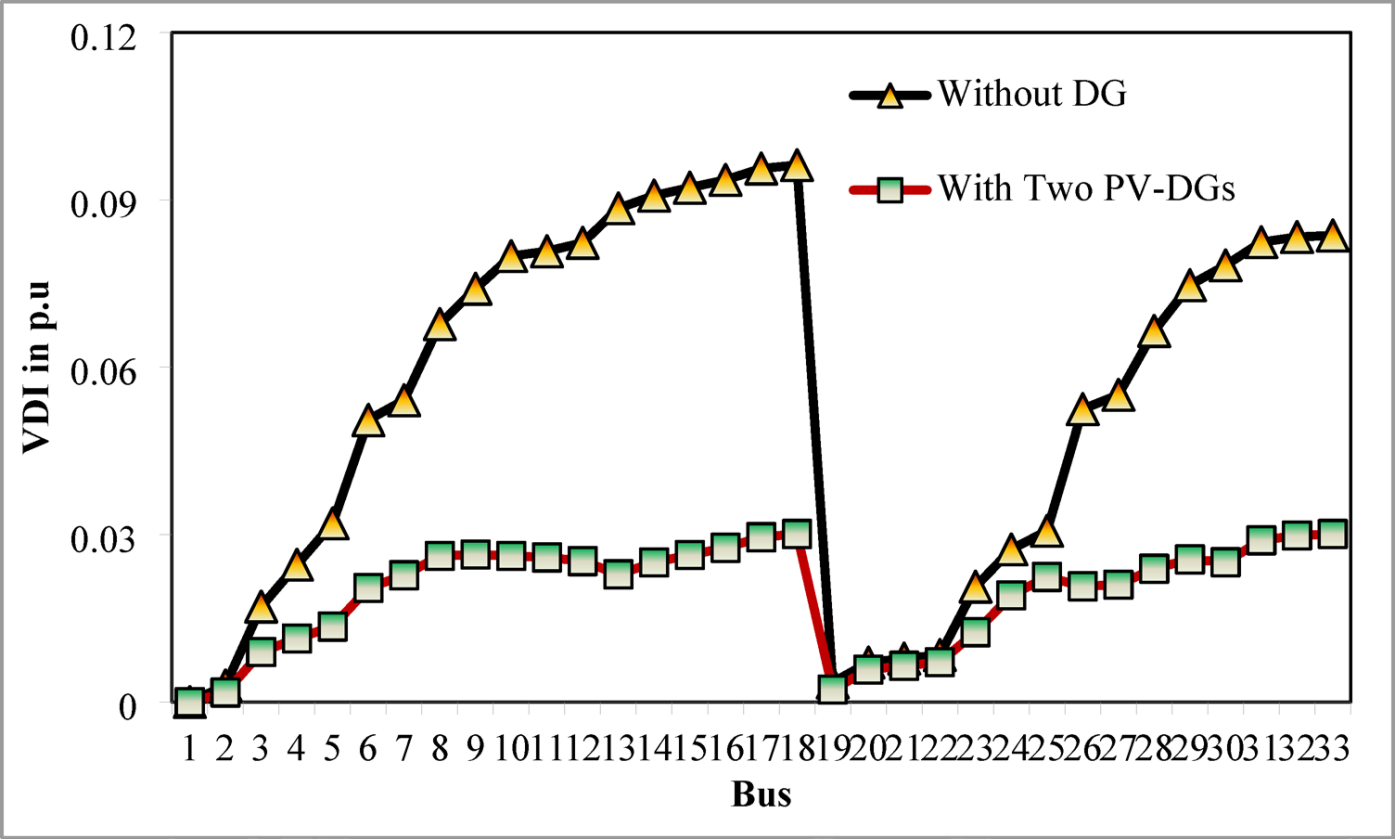
VDI of 33-bus radial DPN after two DG placements.

**Fig. 18:**
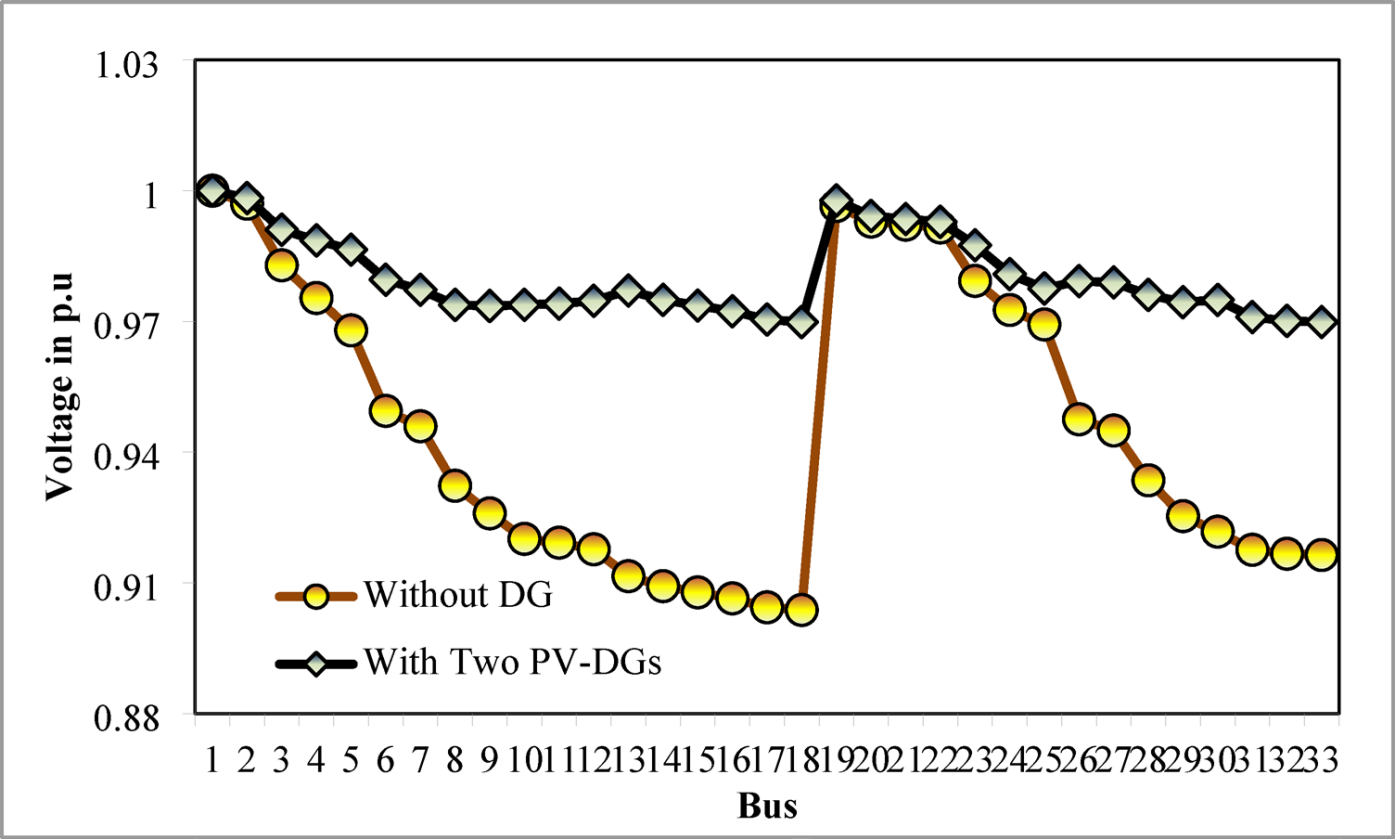
V_bus_ of 33-bus radial DPN after two DG placements.

**Fig. 19:**
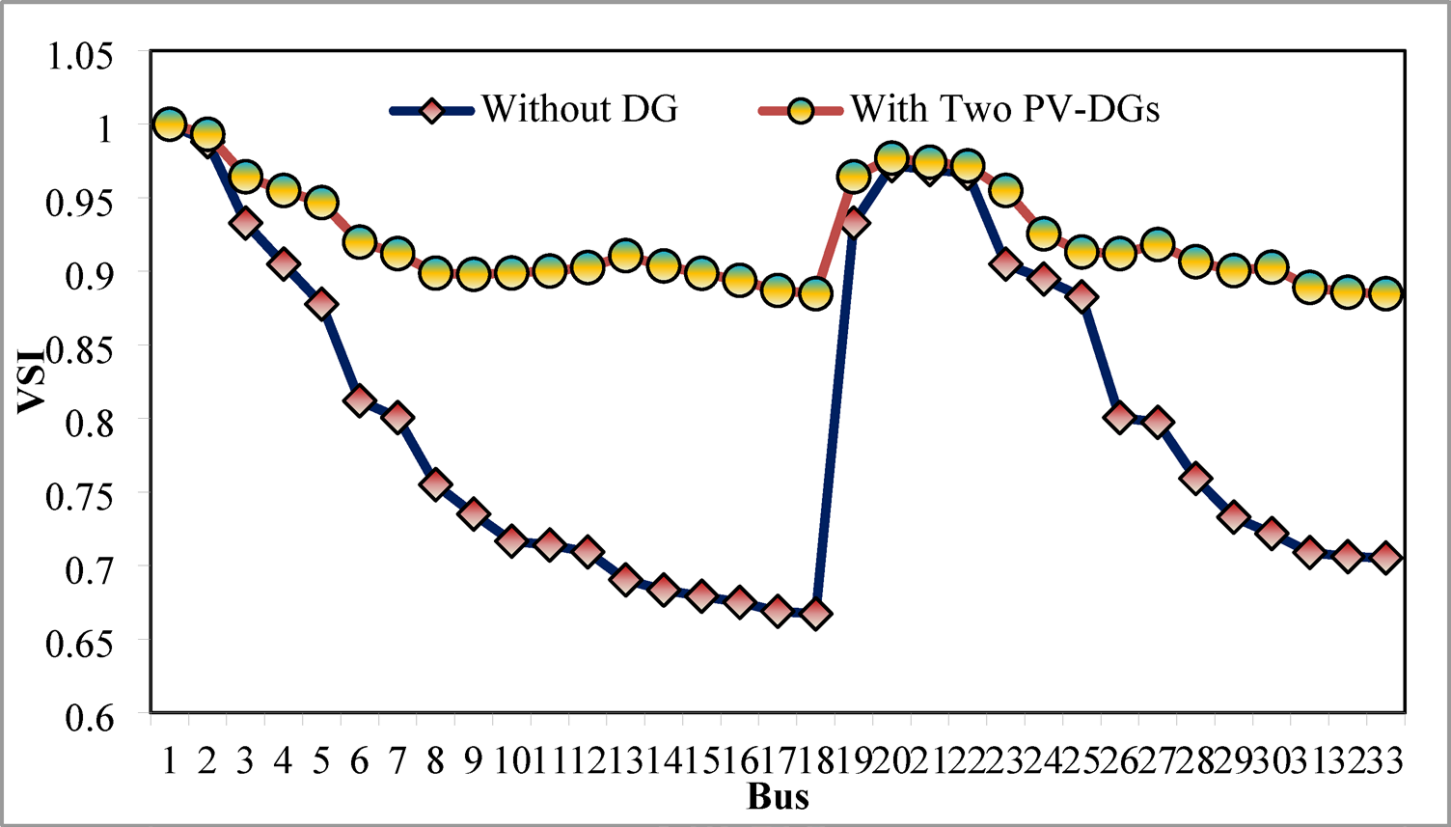
VSI of 33-bus radial DPN after two DG placements.

The simulation findings for the two PV units optimization are presented in Table 5. Figure 14 presents the optimal solution for DG location, size, and fitness value for 50 independent runs. For two DG allocation, JSA optimizes the DGs in the 13th and 30th buses of test system. The best solution is obtained in the 42nd simulation run. The same has been highlighted in the illustration of Fig. 14. The DGs are optimized in the 13th and 30th buses of test system. The JSA converges to an optimal solution for the optimal sizes of 822.8 kW (@ 13th bus) and 1089.5 kW (@ 30th bus). JSA takes only 8 iterations and 29.4 s for convergence. Figure 15 presents the convergence plot of JSA for two units of PV system placements. The total real PL is minimized from 225 kW to 82.99 kW, and VDI_total_ is reduced to 0.6518 p.u. The minimum VDI is 0.0301 p.u. Consequently, the minimum V_bus_ and VSI are increased to 0.9698 p.u. and 0.8848, respectively. Figures 16 and 17 illustrate the simulation results for RPL and VDI without and with two PV system placements, respectively. Likewise, Figs. 18 and 19 present the optimized results for V_bus_ and VSI, respectively. It is evident from the illustrations that the PL and VD are substantially minimized following the optimized integration of two units of PV DG systems.

### Simulation findings with three PV DG placements

**Table 6:** Optimal solution for three PV DG placements.

Parameter	Simulation findings	
	Without DG	With three DGs
Real PL in kW	210.98	69.59
VDI_total_ in p.u	1.8047	0.3293
VDI_min_ in p.u	0.0962	0.0153
V_min_ in p.u	0.9038	0.9846
VSI_min_	0.6671	0.8916
Optimal position(s)	−	13 24 30
Optimal size(s) in kW	−	923.6 1028.3 998.2
Iterations (no.)	−	10
Convergence time (secs)	−	42.3

**Fig. 20:**
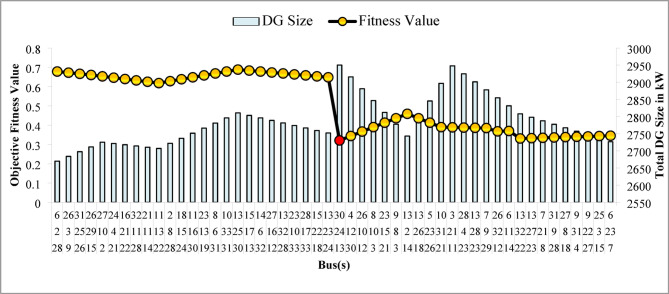
Optimal bus selection for three DGs.

**Fig. 21:**
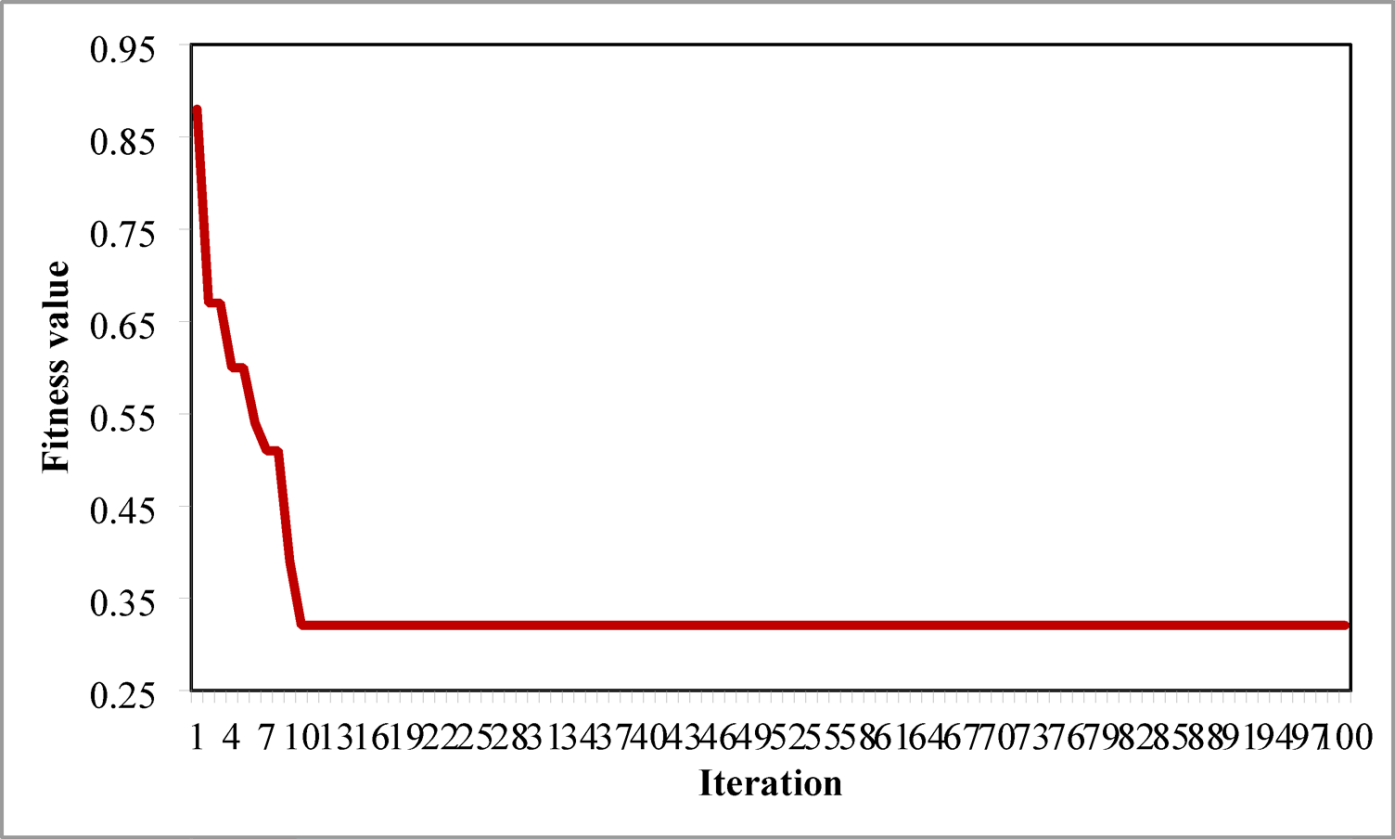
JSA convergence curve for three DG placements.

**Fig. 22:**
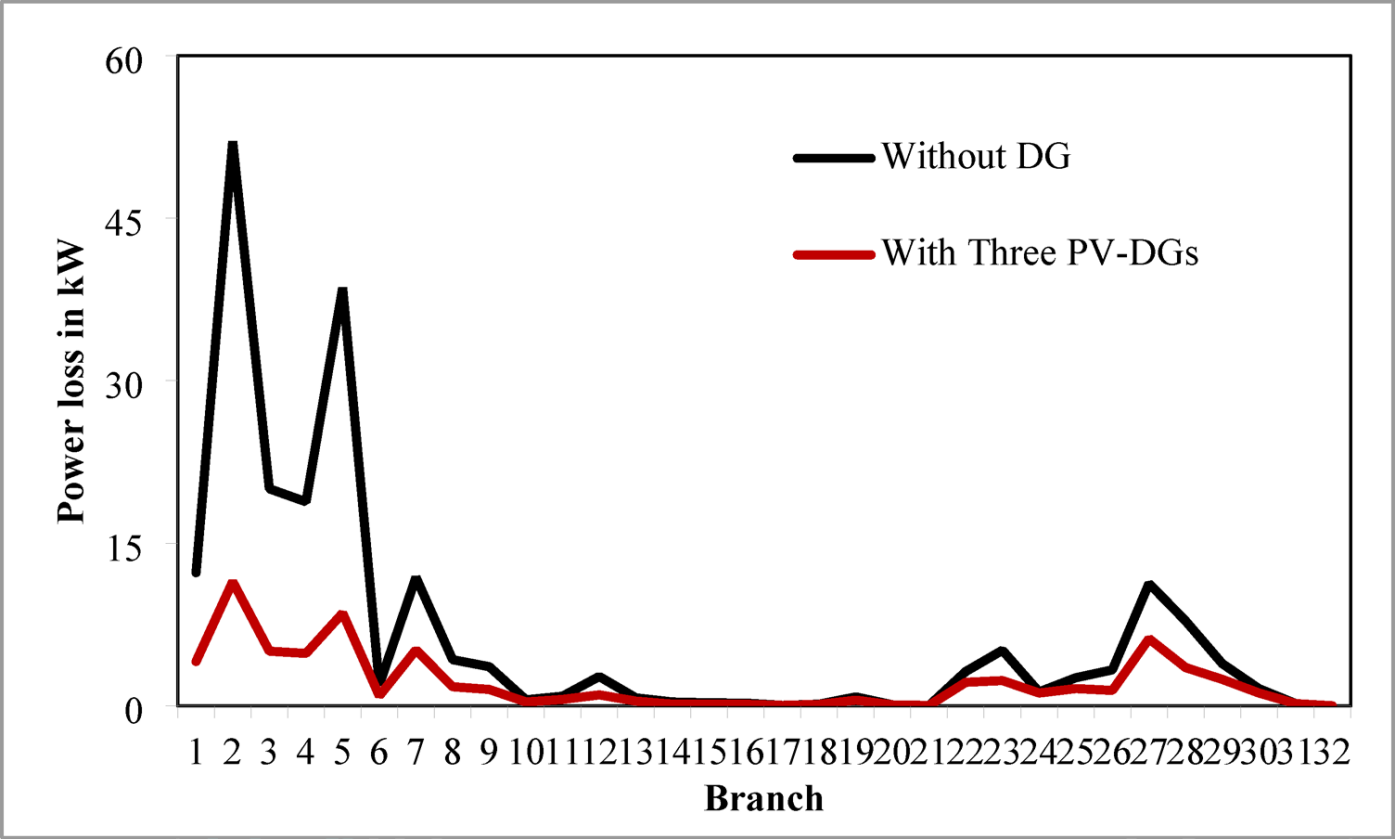
RPL of 33-bus radial DPN for three DG placements.

**Fig. 23:**
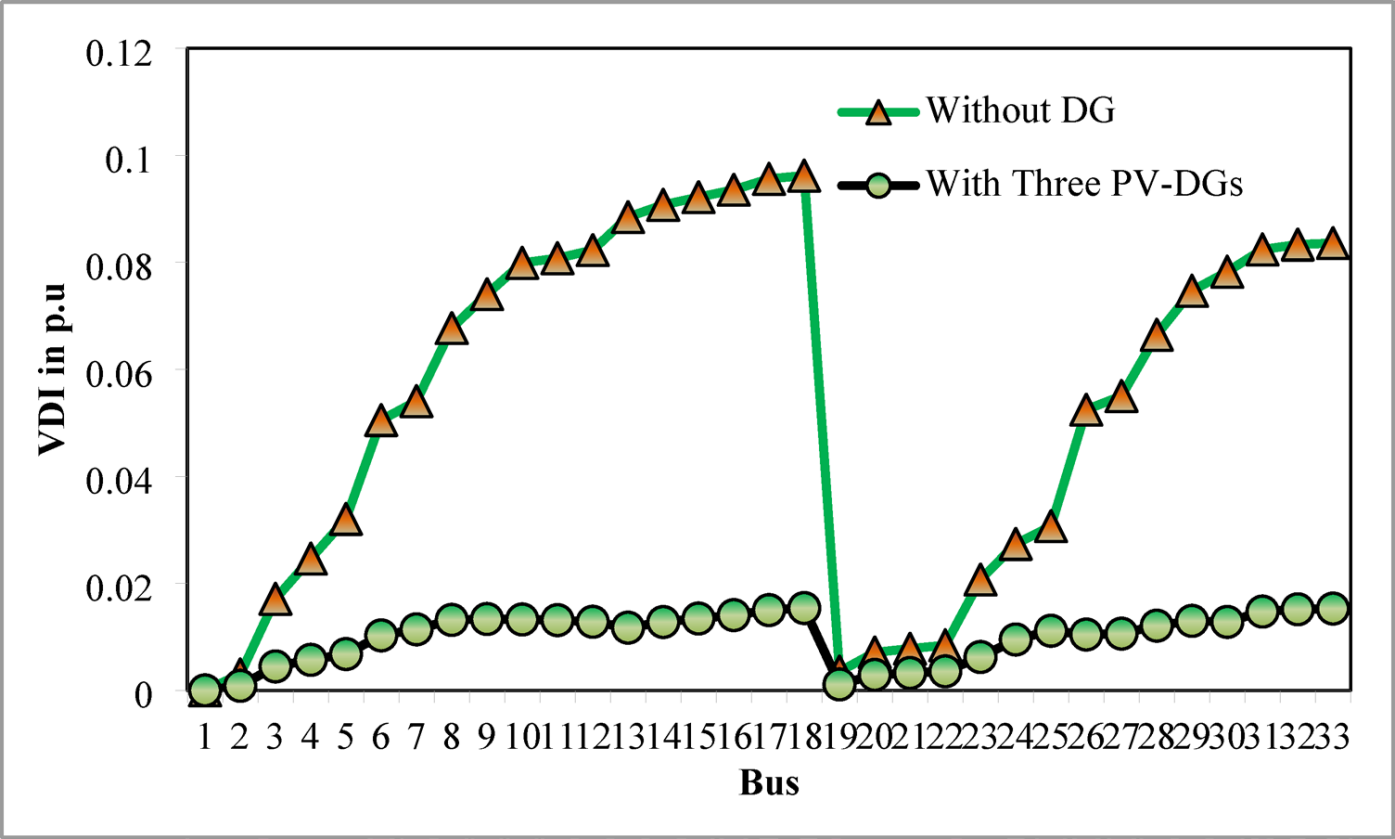
VDI of 33-bus radial DPN for three DG placements.

**Fig. 24:**
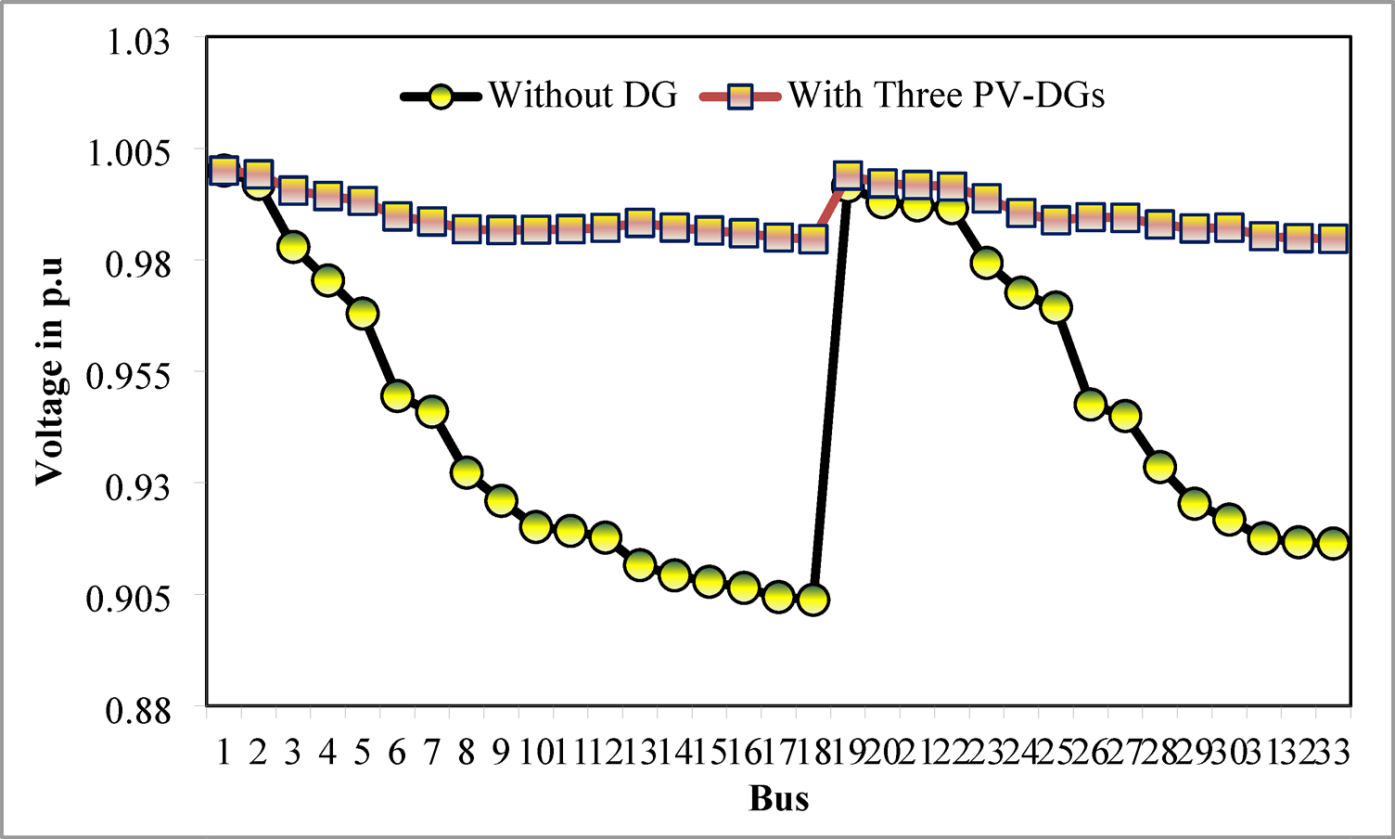
V_bus_ of 33-bus radial DPN for three DGs placements.

**Fig. 25:**
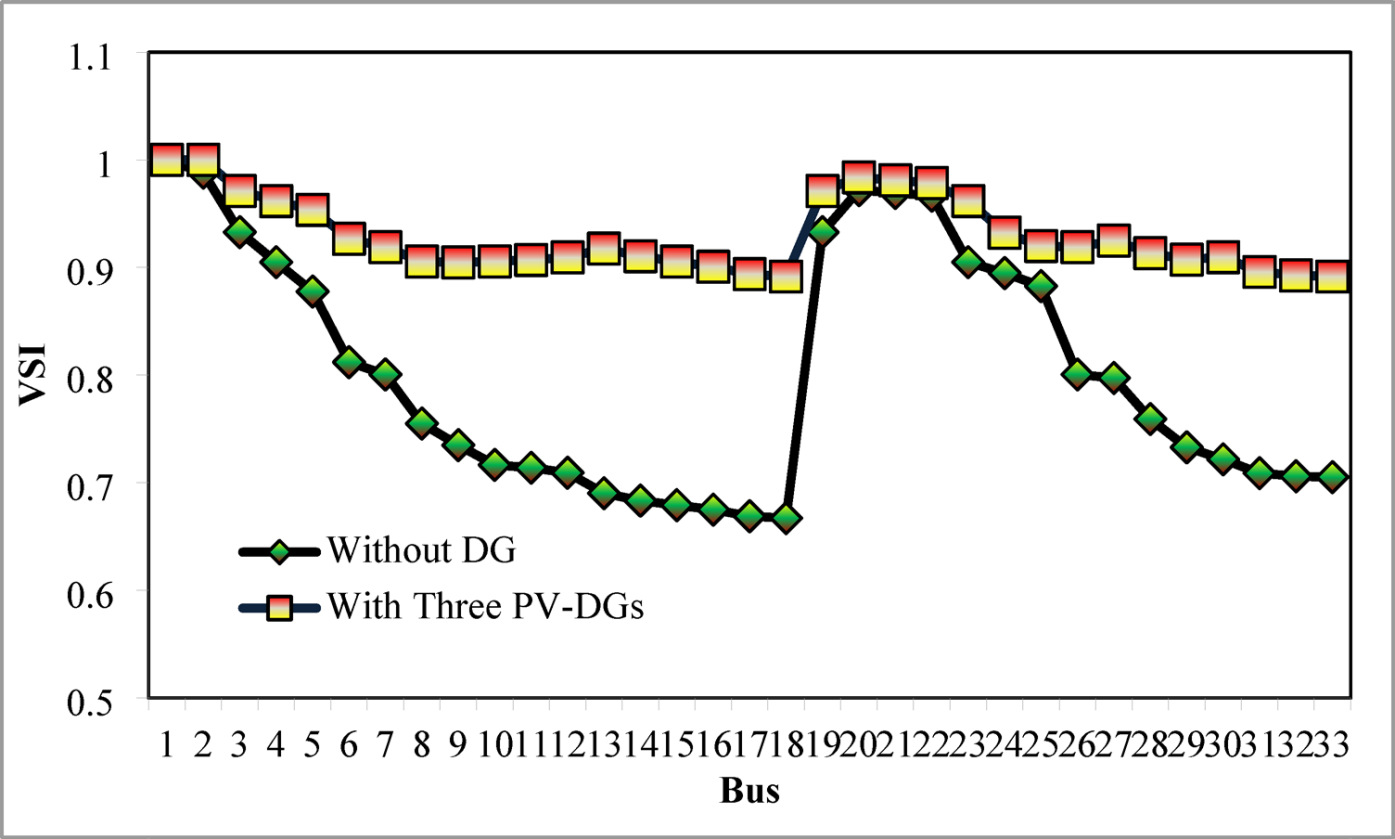
VSI of 33-bus radial DPN for three DG placements.

Table 6 presents the optimized solution for the three DG unit integrations. Figure 20 illustrates the optimal solution for DG location, size, and fitness value for 50 independent runs. For three DG placement case study, JSA optimizes the DGs in the 13th, 24th, and 30th buses. The best solution is reached in the 26th simulation run. The same has been highlighted in the illustration of Fig. 20. JSA converges in 10 iterations and 42.3 s for the optimized PV systems ratings of 923.6 kW, 1028.3 kW, and 998.2 kW, correspondingly, in bus locations 13, 24, and 30. Figure 21 presents the convergence curve of JSA for the optimized three PV system allocations. The optimized inclusion of multiple PV systems reduced the PL to 69.59 kW and VDI_total_ to 0.3293 p.u. Also, the minimum V_bus_ and VSI are increased to 0.9846 p.u. and 0.8916. The optimized simulation outcomes for RPL and VDI are presented in Figs. 22 and 23, respectively. Likewise, Figs. 24 and 25 illustrate the optimized simulation results for V_bus,_ and VSI, respectively.

**Fig. 26:**
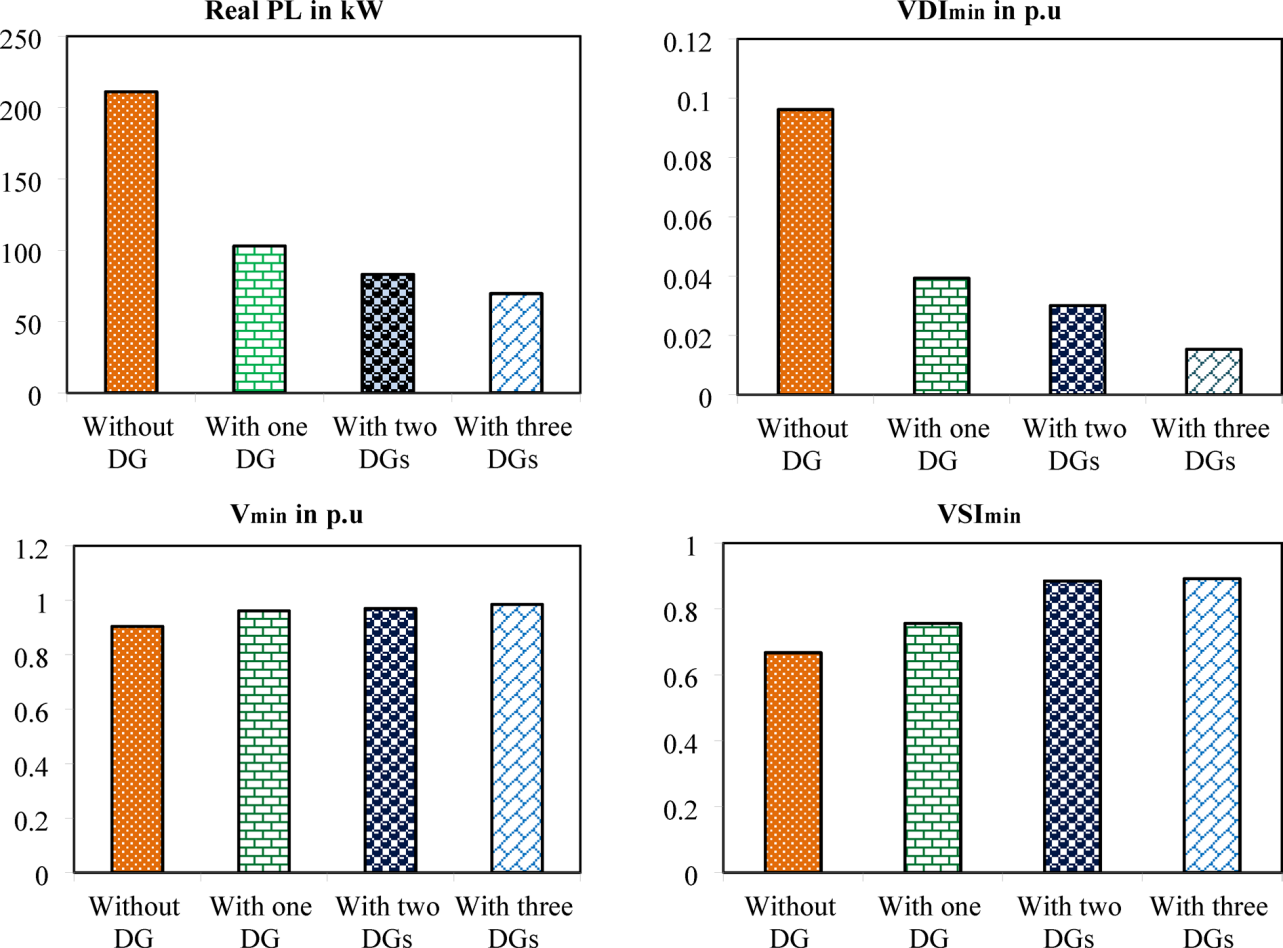
Statistical comparison of simulation results.

### Simulation findings comparison

The simulation findings of different cases of PV DG system placements are graphically summarized in Fig. 26. The total real PL, VDI_total_, VDI_min_, minimum V_bus_, and VSI_min_ of the 33-bus RDPN without DG placement are 210.98 kW, 1.8047 p.u., 0.0962 p.u., 0.9038 p.u., and 0.6671, respectively. The total PL is reduced by 51.23%, 60.66%, and 67.01% for a single, two, and three units of PV system allocations, respectively. VDI_min_ is minimized by 0.0569 p.u., 0.0661 p.u., and 0.0809 p.u. for one, two, and three PV systems placement, respectively. Minimum V_bus_ is increased by 0.0569 p.u. for a single PV, 0.066 p.u. for two PVs, and 0.0808 p.u. for three PV system allocations. And, VSI_min_ is increased by 0.0888, 0.2177, and 0.2245 following a single, two, and three PV systems integration. The simulation outcomes reveal that substantial PL reduction, VP improvement, and VS enhancement have been achieved for all cases of DG placement; however, better results are obtained for three DG placements than two and single DG placements.

Additionally, the effectiveness of the JSA optimization is examined by comparing its simulation findings with other optimization methodologies. Tables 7, 8, 9 present the simulation findings of JSA and other optimization methodologies for single, two, and three PV systems allocation, respectively. The simulation results of LSF-SCA^37^, SCA^37^, BSOA^25^, GA^38^, ALO^24^, TLBO-GWO^32^, WIPSO-GSA^41^, GWO^41^, BFOA^42^, KHA^43^, BA^44^, WOA^45^, and AIS^46^ methodologies are compared to the JSA solutions. For a single DG optimization, JSA results outperform LSF-SCA, SCA, BSOA, GA, ALO, and TLBO-GWO methodologies by giving maximum PL reduction and better V_bus_ enhancement. Likewise, JSA-optimized two DG placements give better PL and VD minimization than LSF-SCA, SCA, BSOA, WIPSO-GSA, and GWO methodologies. Furthermore, JSA-optimized three units of PV systems allocation produced superior outcomes than other methodologies.

**Table 7:** Simulation outcome of JSA and other methodologies for single PV DG placement.

Parameter	Methodology						
	Proposed	LSF-SCA^37^	SCA^37^	BSOA^25^	GA^38^	ALO^24^	TLBO-GWO^32^
Optimal position	6	6	6	8	6	6	6
Optimal size in kW	2334.60	2590.21	2590.15	1857.5	2600	2590.2	1000
RPL in kW	102.89	111.02	111.02	118.12	111.03	111.03	127.28
RPL reduction (%)	51.23	47.38	47.38	NA	47.38	47.38	NA
V_min_ in p.u	0.9607	0.9424	0.9424	0.9441	0.9425	0.9424	0.9285

**Table 8:** Simulation outcome of JSA and other methodologies for two PV DGs placement.

Parameter	Methodology					
	Proposed	LSF-SCA^37^	SCA^37^	BSOA^25^	WIPSO-GSA^41^	GWO^41^
Optimal position(s)	13 60	13 20	13 30	13 31	13 30	13 30
Optimal size(s) in kW	822.8 1089.5	844.84 1166.16	868.28 1136.25	880 924	850 1140	903.04 1201.61
Total size of DGs in kW	1912.3	2011	2004.53	1804	1990	2104.65
RPL in kW	82.99	87.17	87.19	89.34	87.18	87.43
RPL reduction (%)	60.66	58.69	58.68	NA	58.68	NA
V_min_ in p.u	0.9698	0.9683	0.9680	0.9665	NA	0.9706

**Table 9:** Simulation outcome of JSA and other methodologies for three PV DGs placement.

Parameter	Methodology							
	Proposed	LSF-SCA^37^	SCA^37^	BFOA^42^	KHA^43^	BA^44^	WOA^45^	AIS^46^
Optimal position(s)	13 24 30	13 24 30	13 24 30	14 18 32	13 25 30	13 24 30	31 6 14	31 14 3
Optimal size(s) in kW	923.6 1028.3 998.2	805.71 1104.32 1044.22	827.29 1082.15 1022.40	652.1 198.4 1067.2	810 836 841	720 1020 980	748.15 1051.1 650.56	750 750 1500
Total size of DGs in kW	2950.1	2954.26	2931.86	1917.7	2487	2720	2449.81	3000
RPL in kW	69.59	72.79	72.83	89.9	75.41	73.4	77.06	73.62
RPL reduction (%)	67.01	65.50	65.48	57.38	61.12	NA	NA	NA
V_min_ in p.u	0.9846	0.9685	0.9680	0.9705	0.9676	0.9628	0.9686	NA

## Conclusion

This research work successfully employs the Jellyfish Search Algorithm (JSA) for the optimal placement and sizing of solar photovoltaic (PV) distributed generation (DG) units in radial distribution power networks (DPNs). The proposed metaheuristic framework addresses multiple objectives, including real power loss (RPL) minimization, voltage deviation reduction, and voltage stability enhancement, ensuring efficient and reliable grid operation. The methodology is rigorously validated on the IEEE 33-bus radial DPN, demonstrating superior convergence characteristics and solution optimality compared to existing metaheuristic approaches. JSA optimized single PV DG optimized inclusion has cut down the total RPL by 51.23% and minimized the total VDI by 1.2716 p.u with minimum VSI 0.7559. On the other side, total RPL and VDI were reduced by 60.66% and 1.1529 p.u for two units of PV DG optimization. But, after the allocation of three units of PV DG systems, the RPL and total VDI were reduced by 67.01% and 1.4754 p.u. Minimum VSI was increased to 0.8848 and 0.8916 after the optimal inclusion of two and three units of PV DG systems, respectively. Simulation results indicate that JSA-optimized DG placements significantly improve system performance, achieving a substantial reduction in RPL while enhancing voltage stability and overall grid resilience. The comparative study highlights JSA’s superior performance over traditional optimization techniques such as Genetic Algorithm (GA), Particle Swarm Optimization (PSO), and Grey Wolf Optimization (GWO), particularly in handling high-dimensional and nonlinear constraints. Notably, the integration of three PV-DG units yields the highest improvements in network efficiency. Overall, the findings establish JSA as an advanced optimization paradigm for DG placement in modern power systems. Future research may explore the adaptation of JSA for unbalanced distribution networks, real-world practical implementations, and hybridized metaheuristic techniques to further enhance computational efficiency and scalability.

## Data Availability

The datasets used and/or analysed during the current study available from the corresponding author on reasonable request.

## Abbreviations

*P*: Real power flow along a branch
*Q*: Reactive power flow along a branch
*I*: Branch current
*R*: Branch resistance
*X*: Branch reactance
*V*: Voltage
_*Vbus*_: Bus voltage
*P*_*L*_: Real power load
*Q*_*L*_: Reactive power load
*P*_*DG*_: Real power rating of DG
*k*: Number of branches
*n*: Number of buses
*Q*_*DG*_: DG reactive power capacity
*P*_*loss*_: Real power loss
*Q*_*loss*_: Reactive power loss
*Q*_*Tloss*_: Total reactive power losses
*P*_*Tloss*_: Total real power losses without DG optimization
$P_{Tloss}^{DG}$: Total real power losses with DG optimization
*δ*_*1*_*, δ*_*2*_*, and δ*_*3*_: Weightage factors
*f*_*1*_: Objective function for RPL minimization
*f*_*2*_: Objective function for VDI minimization
*f*_*3*_: Objective function for VSI maximization
*V*_*1*_: Nominal bus voltage
*P*_*s*_: Substation real power rating
*N*_*DG*_: Number of DGs
$P_{\min }^{DG}$: Total minimum real power ratings of DGs
$P_{T}^{DG}$: Total optimized real power ratings of DGs
$P_{\max }^{DG}$: Total maximum real power ratings of DGs
*V*_*min*_: Minimum recommended bus voltage
*V*_*max*_: Maximum recommended bus voltage
SOA: Biogeography Based Optimization
VP: Voltage profile
VS: Voltage stability
LSF: Loss sensitivity factor
CSCA: Chaotic sine cosine algorithm
ISOS: Improved oppositional-chaotic symbiotic organisms search
SA: Simulated annealing
GSA: Gravitational search algorithm
BA: Bat algorithm
SCA: Sine cosine algorithm
GA: Genetic algorithm
WOA: Whale optimization algorithm
WIPSO: Weight improved particle swarm optimization
HHO: Harris hawks optimizer
*P*_*pvr*_: Rated real power output
*G*: Actual solar irradiance
*G*_*r*_: Rated solar irradiance
*X*: Logistic chaotic value of jellyfish
*η*: Constant
*ꞵ*: Distribution coefficient
*μ*: Mean position of all jellyfish
*L*_*b*_: Lower boundary of search space
*H*_*b*_: Higher boundary of search space
*γ*: Motion coefficient
_*t*_: Iteration
Iter_max_: Maximum iteration
DG: Distributed generation
DPN: Distribution power network
JSA: Jellyfish search algorithm
PV: Photovoltaic
RPL: Real power loss
WSA: Weighted sum approach
PL: Power losses
TN: Transmission networks
VSI: Voltage stability index
ALO: Ant lion optimization
BSOA: Backtracking search optimization algorithm
EHO: Elephant herding optimization
PSO: Particle swarm optimization
VD: Voltage deviation
SKHA: Stud krill herd algorithm
CSA: Crow search algorithm
OTCDE: Opposition-based tuned-chaotic differential evolution
DE: Differential evolution
AIS: Artificial immune systems
RDPN: Radial distribution power network
PLI: Power loss index
VDI: Voltage deviation index
TCM: Time control mechanism
OC: Ocean current
TCF: _Time control function_
BFS: Backward-forward sweep
MoF: Multi-objective function
BFOA: Bacterial foraging optimization algorithm
KHA: Krill herd algorithm
GWO: Grey wolf optimization
TLBO: Teaching learning based optimization
